# Molecular Engineering of pH-Responsive Anchoring Systems onto Poly(ethylene glycol) Corona

**DOI:** 10.1021/jacs.3c00986

**Published:** 2023-04-19

**Authors:** Shaohua Zhang, Abhinav Srivastava, Wei Li, Sjoerd J. Rijpkema, Vincenzo Carnevale, Michael L. Klein, Daniela A. Wilson

**Affiliations:** † Institute for Molecules and Materials, Radboud University, Nijmegen, 6525 AJ, The Netherlands; ‡ Institute for Computational Molecular Science, Temple University, Philadelphia, Pennsylvania 19122, United States of America; § iGEM-Institute for Genomics and Evolutionary Medicine, Temple University, Philadelphia, Pennsylvania 19122, United States of America

## Abstract

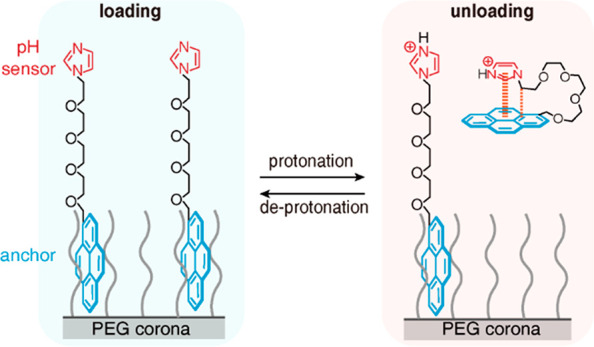

An adaptive surface that can sense and respond to environmental stimuli is integral to smart functional materials. Here, we report pH-responsive anchoring systems onto the poly(ethylene glycol) (PEG) corona of polymer vesicles. The hydrophobic anchor, pyrene, is reversibly inserted into the PEG corona through the reversible protonation of its covalently linked pH-sensing group. Depending on the p*K*_a_ of the sensor, the pH-responsive region is engineered from acidic to neutral and basic conditions. The switchable electrostatic repulsion between the sensors contributes to the responsive anchoring behavior. Our findings provide a new responsive binding chemistry for the creation of smart nanomedicine and a nanoreactor.

Dynamic recruitment of proteins onto a plasma membrane renders cells with spatiotemporally controlled functions.^1^ Adaptive loading of functional entities onto synthetic material is desirable for artificial smart systems.^2−6^ Noncovalent binding by biomolecular recognition and host–guest chemistry provides an effective approach to adaptive loading that overcomes the poor responsiveness and reversibility associated with covalent binding strategy.^7^ Guest molecules of azobenzene,^8−11^ methylviologen,^12−14^ ferrocene,^15−17^ etc. and host molecules of responsive macrocycles^18−23^ and (metal-)organic cages^24−26^ have been developed for stimuli-responsive binding. Although proven successful, the responsiveness relies on the photoisomerization of azobenzene, the redox-state transition of methyl viologen or ferrocene, the structural modification of hosts, or the introduction of competitive binding molecules.^27−29^ Responsive binding based on simple molecular combinations and new molecular mechanisms remains elusive, although it would greatly facilitate the development of smart nanomedicine and nanoreactors.

PEG is a simple and widely used polymer to construct a stealth corona, antifouling coating, and stabilizing ligands on synthetic materials.^30−32^ Recently, we showed that hydrophobic pyrene (Py) can recognize and bind with the PEG corona of the polymer vesicle, which provides a new noncovalent binding strategy.^33^ The distance between the loaded Py is determined by the electrostatic repulsion between its covalently linked organometallic complex. We, therefore, hypothesized that the binding of PEG corona and Py can be made responsive by controlling the electrostatic repulsion between its covalently linked groups (Figure 1a).

**Figure 1:**
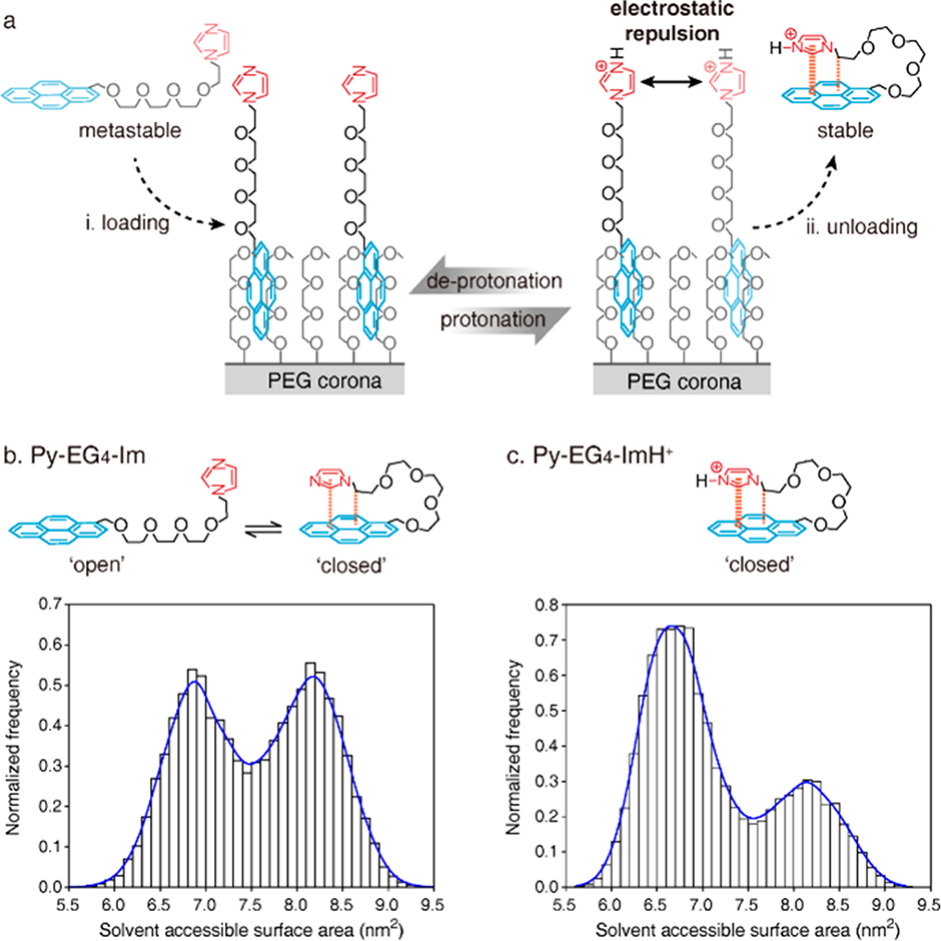
(a) pH-responsive anchoring systems onto PEG corona through the reversible protonation of Im (pH sensor). Conformational distribution for (b) Py-EG_4_-Im and (c) Py-EG_4_-ImH^+^ in water by molecular dynamics simulation. Frequency histograms of solvent accessible surface area for Py-EG_4_-Im and Py-EG_4_-ImH^+^.

To verify our hypothesis, the molecular probe with Py as the anchor, tetraethylene glycol (EG_4_) as the spacer, and imidazole (Im) as the sensing group (Py-EG_4_-Im, Figure 1b) is synthesized. Im (p*K*_a_ = 7.06) can be reversibly protonated to switch on/off the electrostatic repulsion,^34^ which may enable the pH-responsive binding between Py and PEG corona. The stimuli-responsive binding was first evaluated by comparing the stability of Py-EG_4_-Im in water before and after protonation. Through molecular dynamics simulation, Py-EG_4_-Im stays in equilibrium between the open and closed conformations, where the aqueous exposure of Py keeps fluctuating as reflected by the solvent-accessible surface area (Figure 1b and Figure S1). After protonation, Py-EG_4_-ImH^+^ prefers the closed conformation due to the cation−π interaction between ImH^+^ and Py (Figure 1c and Figure S2). The closed conformation would decrease the aqueous exposure of Py, contributing to the higher stability of Py-EG_4_-ImH^+^ than Py-EG_4_-Im in water, as validated by the interaction energy between Py and water (Figure S3). From the thermodynamic point of view, the different aqueous stability suggested that the loaded Py-EG_4_-Im can potentially be released into the solution after protonation (Figure 1a).

The stimuli-responsive binding is further investigated by loading Py-EG_4_-Im onto the PEG corona under different pH conditions (Figures S4 and S5). Here, polymer vesicles assembled with poly(ethylene glycol)-*b*-polystyrene (PEG_44_-*b*-PS_178_) are used to provide a model PEG corona. The glassy PS core provides a stable PEG corona to accommodate Py and withstand centrifugation (Figure S4). The binding of PEG corona and Py is confirmed by comparing the loading of Py-EG_4_-Im on PS and PEG microparticles (Figure S6) and monitoring the fluorescence of Py-EG_4_-Im (Figure S7). The change in Gibbs free energy (Δ*G*) of the binding process is −29.7 kJ/mol, suggesting the thermodynamically favorable binding of the Py and PEG corona (Figure S8). As shown in Figure 2a, the loading amount of Py-EG_4_-Im on the PEG corona diminishes by around 80% when the pH of the loading solution was decreased from 8 to 3. Since the p*K*_a_ of Im is 7.06, we deduce that the pH-dependent loading is caused by the protonation of Im. When the hydroxyl group (OH, p*K*_a_ > 14) is used as the sensing group, the loading of Py-EG_4_-OH is not affected by pH (Figure 2a). Consistently, the fluorescence intensity of Py-EG_4_-Im loaded onto the PEG corona under acidic conditions (pH = 4.3) is lower than that under slightly basic conditions (pH = 7.9), while the fluorescence intensity of Py-EG_4_-OH is not affected (Figure 2b). These results hint that the binding of PEG corona and Py can potentially be responsive when Im is used as the sensing group.

**Figure 2:**
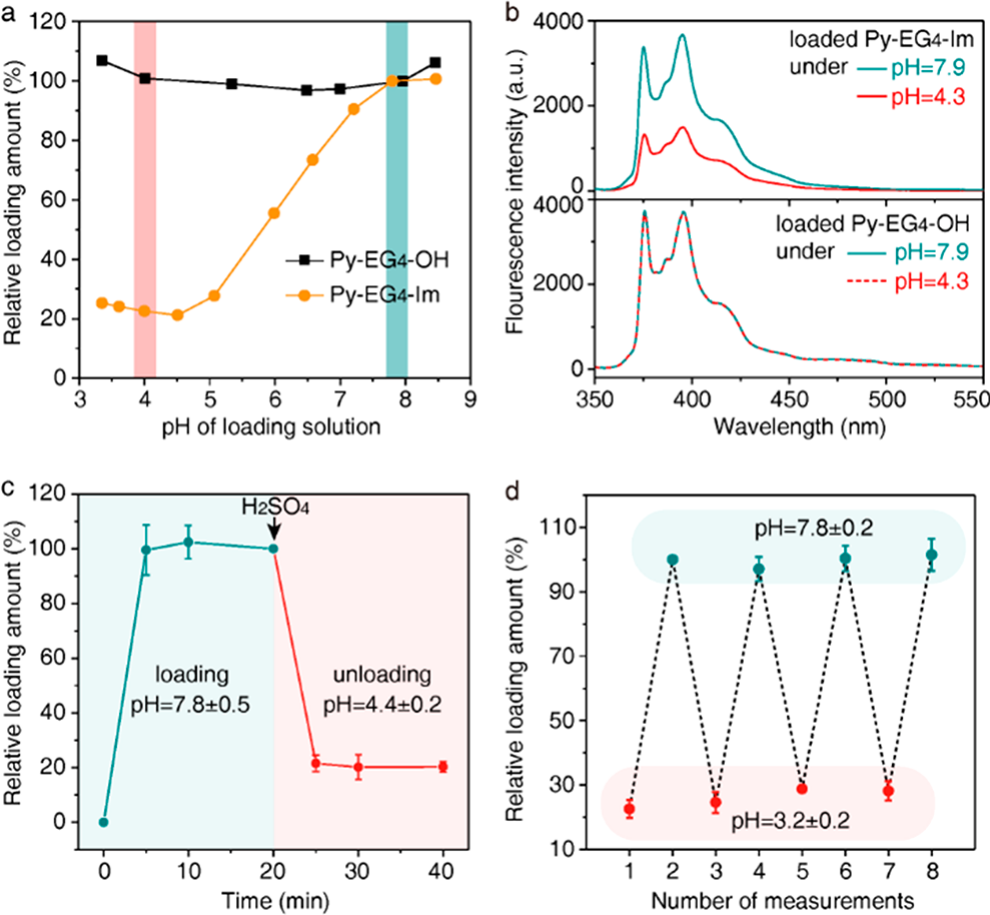
(a) The relative loading amount of Py-EG_4_-Im and Py-EG_4_-OH onto the PEG corona in a loading solution with different pH values. (b) Fluorescence of Py-EG_4_-Im and Py-EG_4_-OH loaded onto the PEG corona. The loading experiments were performed under a pH of 7.9 ± 0.1 (teal line) or 4.3 ± 0.2 (red line). (c) The loading and unloading process of Py-EG_4_-Im onto the PEG corona when the pH value of the solution is switched from 7.8 ± 0.5 to 4.4 ± 0.2. *t* = 5–20 min and 25–40 min are left on purpose to validate the status of the loading and unloading process. Data are the means of triplicate experiments ± SD. (d) The reversible loading and unloading of Py-EG_4_-Im onto the PEG corona by switching the pH value between 3.2 ± 0.2 and 7.8 ± 0.2. Data are the means of duplicate experiments ± SD.

To evaluate the responsiveness, we performed single and multiple loading/unloading cycle(s). As shown in Figure 2c, Py-EG_4_-Im is loaded onto a PEG corona in 5 min under pH = 7.8. When dilute H_2_SO_4_ is added, around 80% of the loaded Py-EG_4_-Im is released into the solution in 5 min under a pH = 4.4. The unloaded Py-EG_4_-Im keeps complete as verified by ^1^H NMR (Figure S9). The single loading/unloading cycle confirms the pH-responsive binding of the Py and PEG corona. Moreover, the multiple loading/unloading cycle of Py-EG_4_-Im is achieved by reversibly changing the pH of the loading solution between 3.2 and 7.8, further validating the excellent pH responsiveness (Figure 2d, Figures S10 and S11). Single and multiple cycling experiments demonstrate that the loaded Py-EG_4_-Im can sense the environmental pH and respond by unloading from the PEG corona. Moreover, the unloading of Py-EG_4_-Im is successfully triggered by endosomal pH, promising the biomedical application of the anchoring systems (Figure S12).

The molecular mechanism for pH-responsive binding is investigated by molecular dynamics simulation and electrostatic shielding experiments. It has been shown by molecular dynamics simulation that Py-EG_4_-Im is loaded by inserting hydrophobic Py into the PEG corona (Figure 1a).^33^ However, the penetration depth of Py-EG_4_-Im in the PEG corona is still not clear, which is important for the loaded Py-EG_4_-Im to sense the environmental pH. When analyzing the PEG corona with the molecular dynamics simulation, the surface exhibits a maximal density of PEG and minimal density of water (gray region), like a “binding pocket” (Figure 3a and Figure S13). During loading, Py would first approach the surface of the PEG corona (<10 ns). After forming a van der Waals interaction with surrounding PEG chains, Py would move around the “binding pocket” of the PEG corona over time (10–100 ns, Figure 3b). The higher PEG density and lower water density of the “binding pocket” should provide the most favorable binding site for Py. Thus, the loaded Py-EG_4_-Im is not penetrating deeper into the PEG corona. Such conformation provides the structural basis for pH-responsive binding.

**Figure 3:**
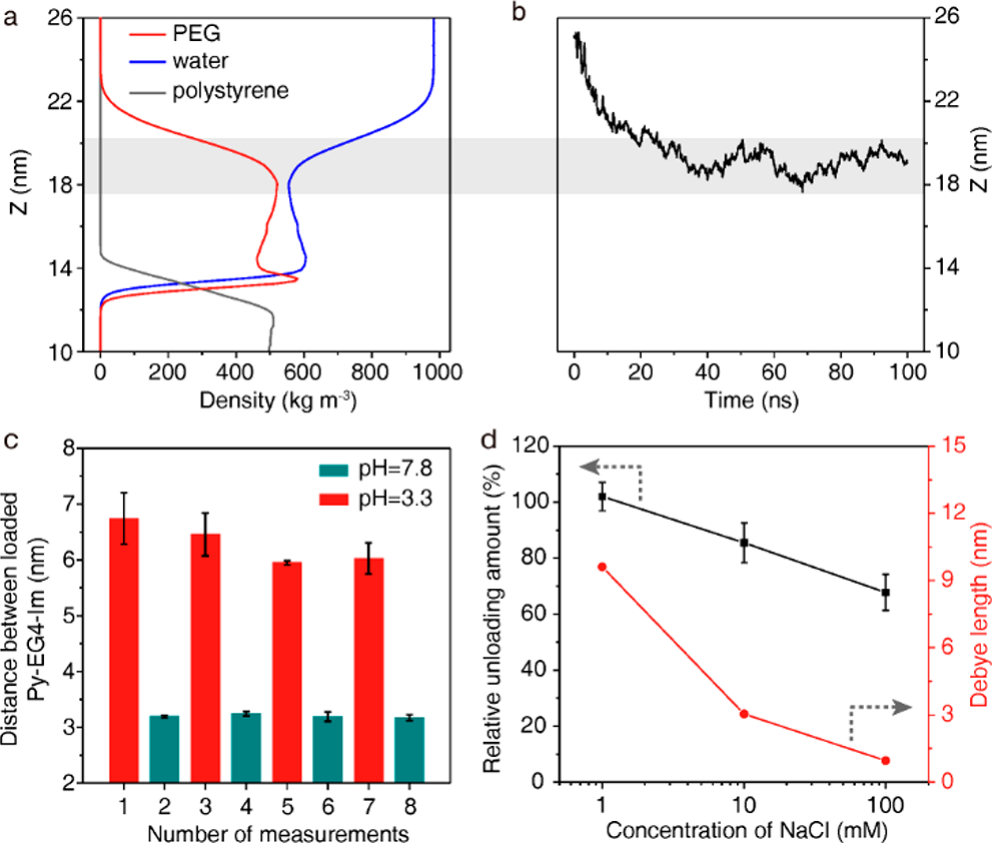
Molecular mechanism of pH-responsive binding. (a) The density of PEG, water, and polystyrene in the monolayer of PEG_44_-*b*-PS_50_. The density plot is averaged over the entire simulation, 0–100 ns. (b) The trajectory of Py-EG_4_-Im in the *z*-axis. The *z* was measured from the center of mass of Py. (c) Distance between the loaded molecular probes on the PEG corona under 7.8 ± 0.2 to 3.3 ± 0.2. Data are the means of duplicate experiments ± SD. (d) The unloading amount of Py-EG_4_-Im in NaCl solution relative to that in 0 mM NaCl (pH = 4.8 ± 0.4). Data are the means of triplicate experiments ± SD.

Furthermore, we calculate the distance between the loaded Py-EG_4_-Im on the PEG corona during multiple loading/unloading cycles. As shown in Figure 3c, the distance is shifted between ∼3 nm and 6–7 nm when the pH of the loading solution is changed between 7.8 ± 0.2 and 3.3 ± 0.2. The over 2-fold increase of the intermolecular distance suggests that the unloading of the molecular probe is driven by the electrostatic repulsion between ImH^+^ (Figure S14). To confirm the role of electrostatic repulsion in pH-responsive binding, we measured the unloading amount of Py-EG_4_-Im in the presence of NaCl (Figure 3d). When the concentration of NaCl is 1 mM, the unloading amount is not affected. This should be caused by the larger Debye length for 1 mM NaCl than the intermolecular distance between loaded Py-EG_4_-Im (9.6 nm vs 3.2 nm), which failed to shield the electrostatic repulsion between ImH^+^. The unloading amount diminishes when 10 mM NaCl with a Debye length smaller than the intermolecular distance is used (3.0 nm). The unloading amount keeps diminishing when NaCl is increased to 100 mM. The salt concentration-dependent unloading of molecular probes validates that the electrostatic repulsion between ImH^+^ contributes to the pH-responsive binding of PEG corona and Py.

Last but not least, we try to manipulate the responsive pH by using sensing groups with different p*K*_a_. Specifically, Im (p*K*_a_ = 7.06) of the molecular probe is replaced with −COOH (p*K*_a_ = 4.76) and −NH_2_ (p*K*_a_ = 9.50), respectively (Figures S15 and S16).^34^ Both Py-EG_4_-COOH and Py-EG_4_-NH_2_ exhibited pH-dependent and pH-responsive loading behavior (Figure 4a, Figure S17, and Figure S18). The pH-responsive region (white region) corresponds well to the p*K*_a_ of the functional group (Figure 4b). This again validates that the pH-dependent probe loading is caused by the (de)protonation of the functional groups. For Py-EG_4_-COOH, −COOH is deprotonated to negatively charged COO^–^ when pH > p*K*_a_, contributing to the decreased loading amount. For Py-EG_4_-NH_2_, −NH_2_ is protonated to positively charged −NH_3_^+^ when pH < p*K*_a_, contributing to the decreased loading amount. The editable responsive pH highlights the potential of the stimuli-responsive binding of Py and the PEG corona for the construction of smart materials.

**Figure 4:**
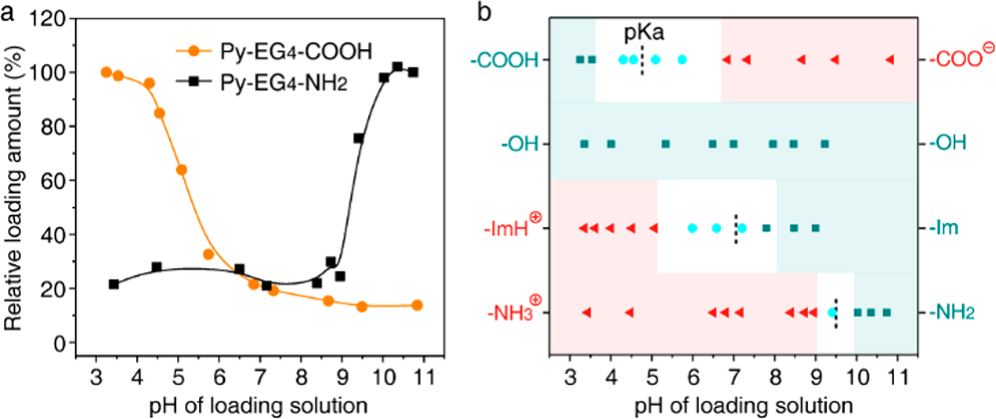
(a) The relative loading amount of Py-EG_4_-COOH and Py-EG_4_-NH_2_ onto PEG corona under different pH values. (b) The change of loading amount as a function of the pH of the loading solution. p*K*_a_ of the pH sensor is marked out by the dashed line. More probes are loaded onto the PEG corona in the teal region than in the red region. The white region is where the loading amount keeps changing.

In summary, we develop a new pH-responsive anchoring system onto the PEG corona. Molecular probes with Py as the anchor; EG_4_ as the spacer; and Im, COOH, or NH_2_ as the pH sensor can be reversibly loaded onto the PEG corona according to the environmental pH. The responsiveness relies on the balance of the attractive force between Py and the PEG corona and the repulsive force between pH sensors. We anticipate that the stimuli-responsive binding with simple molecules would facilitate the design of smart nanomedicine and a nanoreactor.

## Supporting Information Available

Experiment and simulation details, synthesis and characterization of molecular probes, probe-loading, and NMR and MS spectra of molecular probes (PDF)
