# Supplementary material for: Molecular Engineering of pH-Responsive Anchoring Systems onto Poly(ethylene glycol) Corona
Source: J Am Chem Soc. 2023 Apr 19;145(19):10458–62. doi: 10.1021/jacs.3c00986 (PMC10197124; doi:10.1021/jacs.3c00986)
Supplement: Supplementary file 1 — ja3c00986_si_001.pdf [file ja3c00986_si_001.pdf]

Supporting information for

Molecular engineering of pH-responsive anchoring systems onto poly(ethylene glycol) corona

Shaohua Zhang[1], Abhinav Srivastava[2,3], Wei Li[1], Sjoerd J. Rijpkema[1], Vincenzo Carnevale[2,3], Michael L. Klein[2], Daniela A. Wilson[1]*

[1]Institute for Molecules and Materials, Radboud University, Nijmegen, 6525 AJ, The Netherlands;

[2]Institute for Computational Molecular Science, Temple University, Philadelphia, Pennsylvania, PA 19122, USA;

[3]iGEM-Institute for Genomics and Evolutionary Medicine, Temple University, Philadelphia, Pennsylvania, PA 19122, USA

*Corresponding author. Email: d.wilson@science.ru.nl

This PDF file contains:

Table of Contents

1. Materials

2. Instruments

3. Molecular dynamics simulation

4. Synthesis and characterization of molecular probes

5. The loading of molecular probes onto PEG corona

6. Supplementary Figures

7. Supplementary references

8. Characterization of molecular probes

Table of Contents

1. Materials S3

2. Instruments.. S3

3. Molecular dynamics simulation S3

3.1 Conformation of solvated molecular probe S3

3.2 The loading of molecular probe onto PEG corona S3

4. Synthesis and characterization of molecular probes S5

4.1 Synthesis of Py-EG_4-OH S5

4.2 Synthesis of Py-EG_4-Im S5

4.3 Synthesis of Py-EG_4-COOH S7

4.4 Synthesis of Py-EG_4-NH_2 .... S7

5. The loading of molecular probes onto PEG corona S9

6. Supplementary Figures S10

7. Supplementary references.. S30

8. Characterization of molecular probes S33

1. Materials

Tetraethylene glycol, 1-(bromomethyl)pyrene, sodium hydride (NaH, 60% dispersion in mineral oil), *p*-Toluenesulfonyl chloride (TsCl), ethyl bromoacetate, poly(ethylene glycol) diacrylate (PEGDA, M_n=575Da) were purchased from Sigma-Aldrich Chemie (Merck Life sciences N.V.). Cesium carbonate ($CeCO_3$) and imidazole (Im) were obtained from Fluorochem Ltd. Ammonia solution (25%) was obtained from J.T. Baker. Poly(ethylene glycol)-*b*-polystyrene (PEG_{44}-*b*-PS_{178}, PDI=1.01) was synthesized and characterized in our previous paper[1]. PS was purchased from PSS Polymer Standards Service GmbH (M_w=20000Da, PDI=1.02). Poly(ethylene oxide)-*b*-poly(1,2-butadiene) (PEG_{22}-*b*-PBD_{37}, PDI=1.01) was obtained from Polymer Source Inc. Deionized water was produced by Millipore instrument.

2. Instruments

NMR is carried out on a Bruker AVANCE HD nanobay console with a 9.4 T Ascend magnet (400 MHz). Chemical shifts are given in parts per million (ppm) to tetramethylsilane (TMS, δ 0.00 ppm), which is used as the internal standard. Coupling constants are reported as J-values in Hz. Mass spectra are obtained with JEOL AccuTOF CS JMS-T100CS by dissolving molecular probes in methanol. The absorbance of molecular probes is measured with a UV-vis spectrophotometer (JASCO V-630). TEM is obtained with JEOL JEM-1400 FLASH. Fluorescence is measured with Spectrofluorometer (JASCO FP-8300ST). The size of polymer vesicles is measured with Malvern DLS-Zetasizer. The concentration of polymer vesicles is measured with NanoSight LM10. Isothermal titration calorimetry is measured with AutoiTC200. Fluorescent microscopy images are obtained with Leica DMi8 widefield microscope (excitation: 395 nm).

3. Molecular dynamics simulation

3.1 Conformation of solvated molecular probe

Py-EG_4-Im and Py-EG_4-ImH^+ are constructed with a software package of PACKMOL[2]. As-constructed probes are solvated by water molecules. CHARMM36 all-atom force fields are used for Py-EG_4-Im and Py-EG_4-ImH^+. VMD is used for the GROMACS run input topology for Py-EG_4-Im and Py-EG_4-ImH^+[3,4]. An initial energy minimization and NPT equilibration (100 ns) are implemented for Py-EG_4-Im and Py-EG_4-ImH^+.

3.2 The loading of molecular probe onto PEG corona

PEG corona is constructed with PEG_{44}-*b*-PS_{50} using CHARMM-GUI interface (Polymer Builder feature)[5,6]. The types, charges, and parameters of atom are assigned with the program of CHARMM General Force Field[7-10]. PEG_{44}-*b*-PS_{50} is configured with CHARMM36 all-atom force fields[11]. A configuration of 400 polymers is constructed by replicating PEG_{44}-*b*-PS_{50} for 20 times along X and Y direction. The configuration is energy minimized with steepest descent method (10 fs time steps, 5000 steps). An NPT equilibration is implemented (10 ns, 2 fs time steps). Temperature (283 K) and pressure (1 bar) are maintained with velocity rescaling method and semi-isotropic pressure coupling by Berensden method (coupling constant: 5 ps), respectively [12,13]. Coulombic and van der Waals interaction are cutoff at 1 nm. Particle mesh Ewald summation (grid size: 0.12 nm) is used to correct long-range interactions[14-16]. Hydrogen bond is constrained with LINCS algorithm[17]. Periodic boundary condition is used for all directions. As-equilibrated configuration is fully hydrated with 123825 water molecules by SPC water model[18,19]. An NPT equilibration for the solvated configuration is implemented (100 ns, 2 fs time steps).

For molecular probe, the same set of run input parameters is used as described in section 3.1. After equilibration, molecule probe in the most stable configuration is loaded onto the solvated PEG corona. The configuration of loaded probe is allowed to energy minimize by NPT equilibration (100 ns). The simulations are performed with a software package of GROMACS-2020.3[20-26].

4. Synthesis and characterization of molecular probes

4.1 Synthesis of Py-EG$_4$-OH (1)

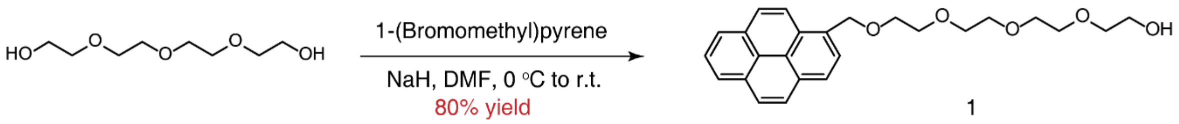

Tetraethylene glycol (970 mg, 5.0 mmol, 1 eq.) was dissolved in DMF (10 mL), which was cooled to 0 °C. NaH (120 mg, 3.0 mmol, 0.6 eq.) was added. After stirring for 30 min under 0 °C, 1-(bromomethyl)pyrene (295 mg, 1.0 mmol, 0.2 eq.) was added. The resulting mixture was stirred for 1 h under 0 °C, and then for 16 h under room temperature (~21 °C). The reaction was quenched through the slow addition of methanol (1 mL) and then water (20 mL). The mixture was extracted with dichloromethane (DCM, 3 ×25 mL). The combined organic layer was washed with saturated $NaHCO_3$ and saturated brine, and dried with Na_2SO_4. The solid was filtered off, and the filtrate was concentrated by rotary evaporation. The product was purified by column chromatography (gradient, DCM → AcOEt). **1** was obtained as a viscous light-brown oil (326 mg, 80%).

^{1}H NMR (400 MHz, $CDCl_3$, δ) 8.41 (d, ^3J = 9.3 Hz, 1H, Py*H*), 7.93-8.23 (m, 8H, Py*H*), 5.28 (s, 2H, Py-C*H*$_2$-O), 3.50–3.80 (m, 16H, (C*H*$_2$-C*H*$_2$-O)$_4$). **^{13}C NMR** (101 MHz, $CDCl_3$, δ): 131.4, 131.3, 131.3, 130.8, 129.4, 127.7, 127.4, 127.1, 125.9, 125.2, 125.0, 124.7, 124.5, 123.6, 72.5, 71.9, 70.7, 70.6, 70.6, 70.6, 70.3, 69.5, 61.7. **HR-MS** (ESI, positive, methanol) (m/z): [M + Na]$^+$ Calcd for $C_{25}H_{28}O_5Na$, 431.183; Found, 431.183.

4.2 Synthesis of Py-EG$_4$-Im (3)

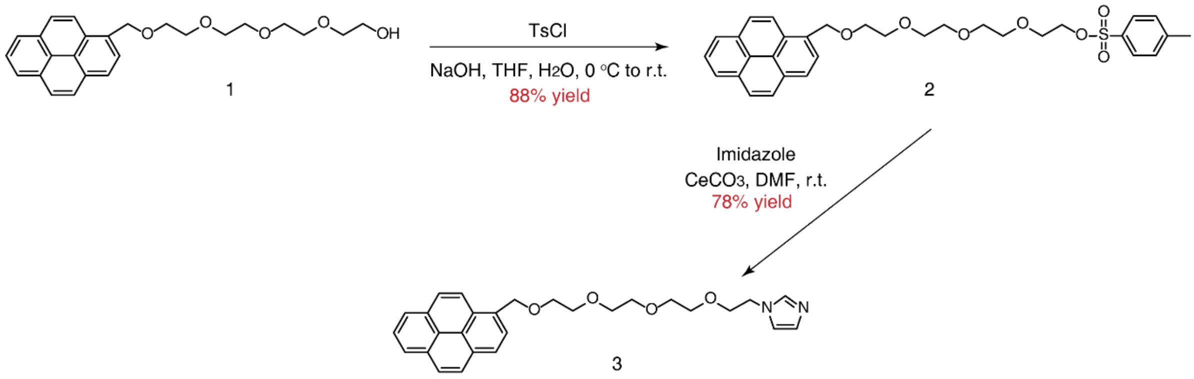

Synthesis of Py-EG$_4$-Ts (2)

Py-EG$_4$-OH (314 mg, 0.77 mmol, 1 eq.) was dissolved in THF (2 mL), which was cooled to 0 °C. NaOH (123 mg, 3.08 mmol, 4 eq.) in water (0.68 mL) was added. After stirring for 20 min at 0 °C, TsCl (220 mg, 1.16 mmol, 1.5 eq.) in THF (2 mL) was added. The resulting mixture was stirred for 2 h under 0 °C, and then for 20 h under room temperature (~21 °C). The mixture was poured into water (10 mL), which was extracted with DCM (3×20 mL). The combined organic layer was washed with water (3×20 mL), and dried with Na_2SO_4. The solid was filtered off, and the filtrate was concentrated by rotary evaporation. The product was purified by column chromatography (gradient, DCM → DCM/AcOEt, 9:1 v/v). **2** was obtained as a viscous and yellowish oil (380 mg, 88%).

^{1}H NMR (400 MHz, $CDCl_3$, δ): 8.40 (d, ^3J = 9.3 Hz, 1H, Py*H*), 7.98-8.23 (m, 8H, Py*H*), 7.75 (d, ^3J = 8.3 Hz, 2H, $C_{arom}H$-C-SO_3), 7.27 (2H, $C_{arom}H$-C-CH_3), 5.28 (s, 2H, Py-CH_2-O), 4.10 (t, ^3J = 4.9 Hz, 2H, CH_2-SO_3), 3.47–3.79 (m, 14H, (CH_2-CH_2-O)$_3$), 2.38 (s, 3H, -CH_3). **^{13}C NMR** (101 MHz, $CDCl_3$, δ): 144.7, 133.0, 131.4, 131.3, 131.3, 130.8, 129.8, 129.4, 128.0, 127.7, 127.4, 127.4, 127.1, 126.0, 125.2, 125.0, 124.7, 124.5, 123.6, 71.9, 70.8, 70.7, 70.6, 70.5, 69.6, 69.2, 68.6, 21.6. **HR-MS** (ESI, positive, methanol) (m/z): $[M + Na]^+$ Calcd for $C_{32}H_{34}O_7SNa$, 585.192; Found, 585.190.

Synthesis of Py-EG$_4$-Im (3)

Py-EG$_4$-Ts (200 mg, 0.36 mmol, 1 eq.), imidazole (22 mg, 0.32 mmol, 0.9 eq.), and $CeCO_3$ (313 mg, 0.96 mmol, 2.7 eq.) were dissolved in DMF (5 mL), which was stirred for 24 h under room temperature (~21 °C). The mixture was diluted with DCM（27 mL), and washed with saturated $NaHCO_3$ (2×20 mL) and saturated brine (2×20 mL). The organic layer was dried over Na_2SO_4, and concentrated by rotary evaporation. The product was purified by column chromatography (gradient, DCM → DCM/MeOH, 96:4 v/v). **3** was obtained as a viscous and yellowish oil (128 mg, 78%).

^{1}H NMR (400 MHz, $CDCl_3$, δ): 8.40 (d, *J* = 9.3 Hz, 1H, Py*H*), 7.97–8.23 (m, 8H, Py*H*), 7.46 (s, 1H, Im*H*), 7.01 (s, 1H, Im*H*), 6.90 (s, 1H, Im*H*), 5.28 (s, 2H, Py-CH_2-O), 3.96 (t, *J* = 5.2 Hz, 2H, CH_2-N), 3.44–3.80 (m, 14H, (CH_2-CH_2-O)$_3$). **^{13}C NMR** (101 MHz, $CDCl_3$, δ): 137.5, 131.4, 131.3, 131.3, 130.8, 129.4, 129.2, 127.7, 127.4, 127.1, 126.0, 125.2, 125.2, 124.9, 124.7, 124.5, 123.6, 119.4, 71.9, 70.8, 70.7, 70.6, 70.5, 70.4, 69.6, 46.9. **HR-MS** (ESI, positive, methanol) (m/z): $[M + H]^+$ Calcd for $C_{28}H_{31}O_4N_2$, 459.228; Found, 459.228.

4.3 Synthesis of Py-EG4-COOH (4)

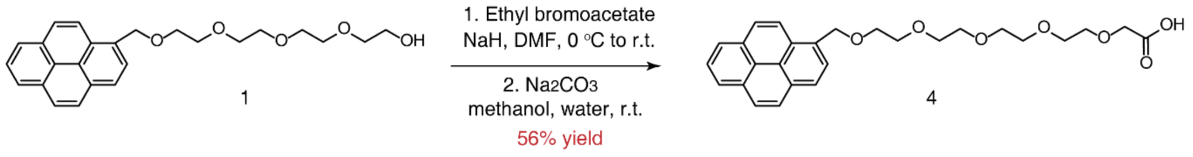

Py-EG4-OH (140 mg, 0.34 mmol, 1 eq.) was dissolved in 2 mL DMF and cooled to 0 °C. NaH (48 mg, 1.20 mmol, 3.5 eq.) was added to the above solution, which was incubated for 30 min under 0 °C. Ethyl bromoacetate (284 mg, 1.70 mmol, 5 eq) was added dropwise. The mixture was stirred for 1 h under 0 °C, and stirred for 16 h under 21 °C. The reaction was quenched by the slow addition of methanol and water. As-obtained solution was extracted with DCM, and was washed with saturated $NaHCO_3$ 2x and saturated NaCl 1x. After being dried by Na_2SO_4 and concentrated by rotary evaporation, the crude intermediate was dissolved in a mixed solvent of 1 mL methanol and 1 mL water, to which Na_2CO_3 (43 mg, 0.41 mmol, 1.2 eq.) was added. After stirring for 30 min, concentrated HCl was added to adjust the pH to the 3-4 range. The solution was extracted by DCM and dried by Na_2SO_4. The product was purified by column chromatography (gradient, DCM → DCM/MeOH, 9:1 v/v). **4** was obtained as a viscous oil (88 mg, 56%).

^{1}H NMR (400 MHz, $CDCl_3$, δ): 8.34 (d, J = 9.3 Hz, 1H, Py*H*), 7.92–8.19 (m, 8H, Py*H*), 5.22 (s, 2H, Py-CH_2-O), 3.89 (s, 2H, OCH_2-COOH), 3.33–3.73 (m, 16H, (CH_2-CH_2-O)$_4$). **^{13}C NMR** (101 MHz, $CDCl_3$, δ): 131.3, 131.2, 130.8, 129.4, 127.7, 127.4, 127.4, 127.2, 126.0, 125.2, 125.2, 124.9, 124.7, 124.5, 123.5, 71.7, 70.3, 70.1, 70.0, 69.8, 69.7, 69.6, 69.5, 69.1. **HR-MS** (ESI, positive, methanol) (m/z): $[M + Na]^+$ Calcd for $C_{27}H_{30}O_7Na$, 489.189; Found, 489.188.

4.4 Synthesis of Py-EG4-NH2 (5)

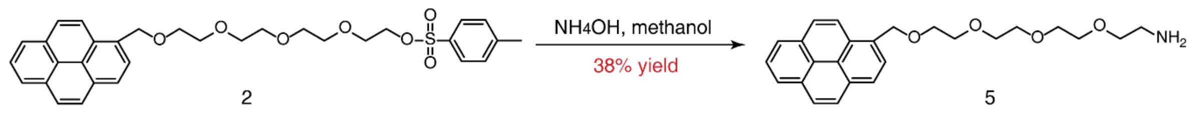

Py-EG4-NH2 was synthesized through a modified procedure[27]. Py-EG4-Ts (30 mg, 0.065 mmol) was dissolved in 2 mL methanol and 2 mL ammonia solution. The mixture was stirred for 7 days under room temperature (~21 °C). 1 mL ammonia was added every 24 h. The mixture was extracted with chloroform. The combined organic layer was dried over Na_2SO_4, and concentrated by rotary evaporation. The product was purified by column chromatography

(gradient, chloroform → chloroform/MeOH, 9:1 v/v). **5** was obtained as a viscous oil (10 mg, 38%).

^{1}H NMR (400 MHz, $CDCl_3$, δ): 8.41 (d, *J* = 9.3 Hz, 1H, Py*H*), 7.97–8.26 (m, 8H, Py*H*), 5.29 (s, 2H, Py-C*H*$_2$-O), 3.53–3.81 (m, 12H, PEG backbone), 3.42 (t, *J* = 5.2 Hz, 2H, -C*H*$_2$-CH_2-NH_2), 2.76 (t, *J* = 5.2 Hz, 2H, -CH_2-C*H*$_2$-NH_2). **^{13}C NMR** (101 MHz, $CDCl_3$, δ): 131.3, 130.8, 129.4, 127.7, 127.4, 127.4, 127.1, 126.0, 125.2, 125.0, 124.7, 124.5, 123.6, 73.1, 71.9, 70.7, 70.7, 70.6, 70.5, 70.3, 69.6, 41.6. **HR-MS** (ESI, positive, methanol) (m/z): $[M + H]^+$ Calcd for $C_{25}H_{30}O_4N$, 408.217; Found, 408.217.

5. The loading of molecular probes onto PEG corona

5.1 Preparation of polymer vesicles with PEG corona

Polymer vesicles were prepared by solvent switch method. Briefly, 10 mg PEG_{44}-*b*-PS_{178} was dissolved in 1 mL mixture of 1,4-dioxane and THF (1:4 v/v). After stirring for 0.5 h, water was injected (1 mL/h, 1 mL). As-obtained polymer vesicles were quenched by adding 10 mL water. After centrifugation and water washing (3 times), the polymer vesicles were dispersed in water. The polymer vesicles were evenly divided into 10 portions for the loading experiments. NanoSight LM10 was used to measure the concentration of polymer vesicles.

5.2 The loading of molecular probes onto PEG corona under different pH

Molecular probes of Py-EG_4-OH, Py-EG_4-Im, Py-EG_4-COOH, and Py-EG_4-NH_2 were dissolved in water. The probe solution was added to the dispersion of polymer vesicles under different pH values. The pH was adjusted by adding H_2SO_4 or NaOH. In the loading solution, the probe concentration was around 20 uM, and the concentration of polymer vesicles is 2.72×10^{-4} μM. After incubating under room temperature for 10 min, the polymer vesicles were removed by centrifugation. The supernatant was used for UV-vis absorbance measurements. As-obtained polymer vesicles were redispersed in 1 mL water for fluorescence measurements.

5.3 Loading and unloading of molecular probes

A molecular probe, for instance, Py-EG_4-Im, was loaded onto polymer vesicles according to section 5.2. H_2SO_4 was added into the loading solution to induce the unloading of molecular probes. For multiple loading and unloading cycles, H_2SO_4 and NaOH were alternatively added into the loading solution to adjust the pH. The distance between loaded probes is calculated according to the loading amount and size of the polymer vesicles[1].

6. Supplementary Figures

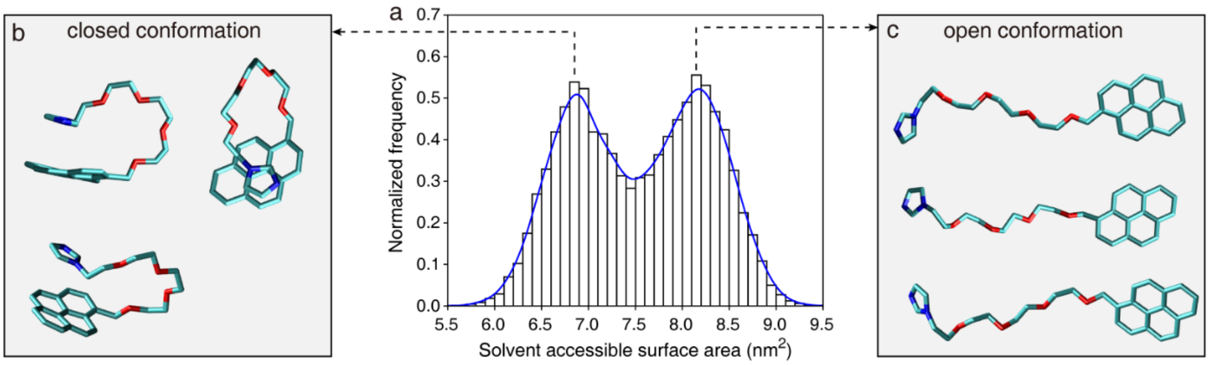

Figure S1. Conformation distribution of Py-EG4-Im dissolved in water by molecular dynamics simulation. (**a**) Frequency histograms of solvent accessible surface area for Py-EG4-Im. (**b**, **c**) The most frequent conformations of Py-EG4-Im. Py-EG4-Im is shown as the framework model: cyan, C; red O; blue N.

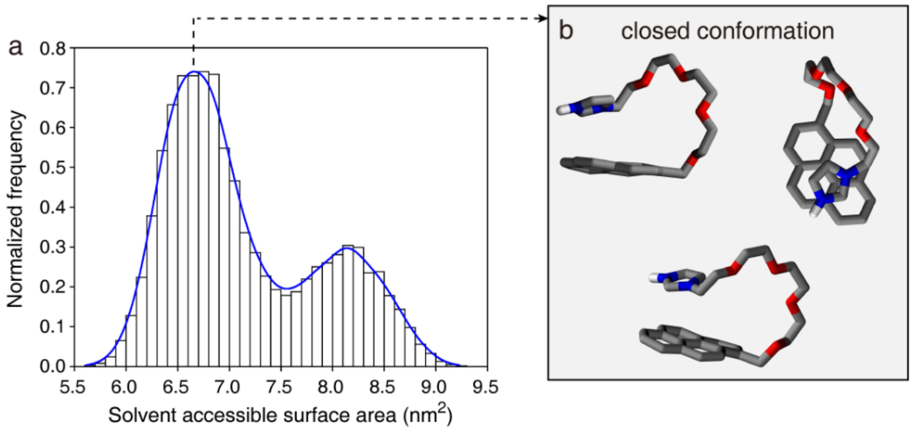

Figure S2. Conformation distribution of Py-EG4-ImH+ dissolved in water by molecular dynamics simulation. (**a**) Frequency histograms of solvent accessible surface area for Py-EG4-ImH+. (**b**) The most frequent conformation of Py-EG4-ImH+. Py-EG4-ImH+ is shown as the framework model: grey C; red O; blue N; white H.

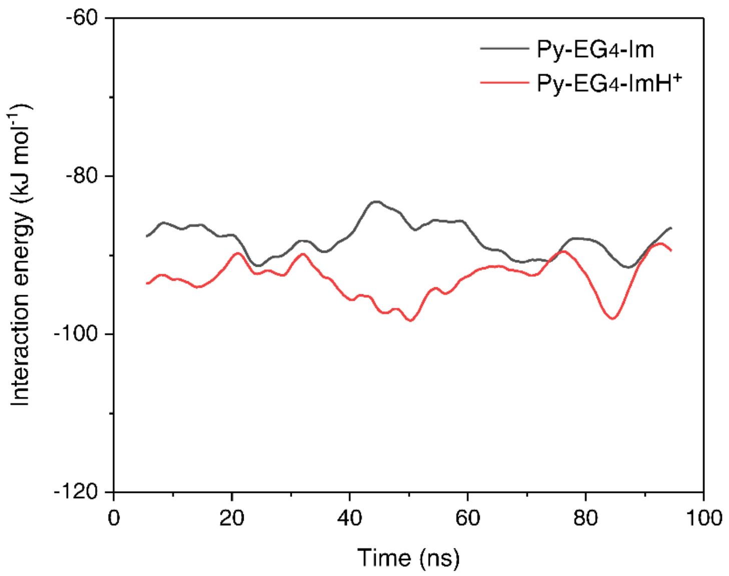

Figure S3. The interaction energy of Py and water for Py-EG4-Im and Py-EG4-ImH$^+$ by molecular dynamics simulation. The interaction energy is the potential energy between solute and solvent molecule, which is the sum of electrostatic and van der Waals interaction. The potential energy for electrostatic interaction is calculated *via* Coulombic interactions, and for van der Waals interaction is calculated *via* Lennard Jones interactions. The interaction energy quantifies the strength of interaction between a solute and solvent, which is the energy released when one mole of Py interacts with one mole of water.

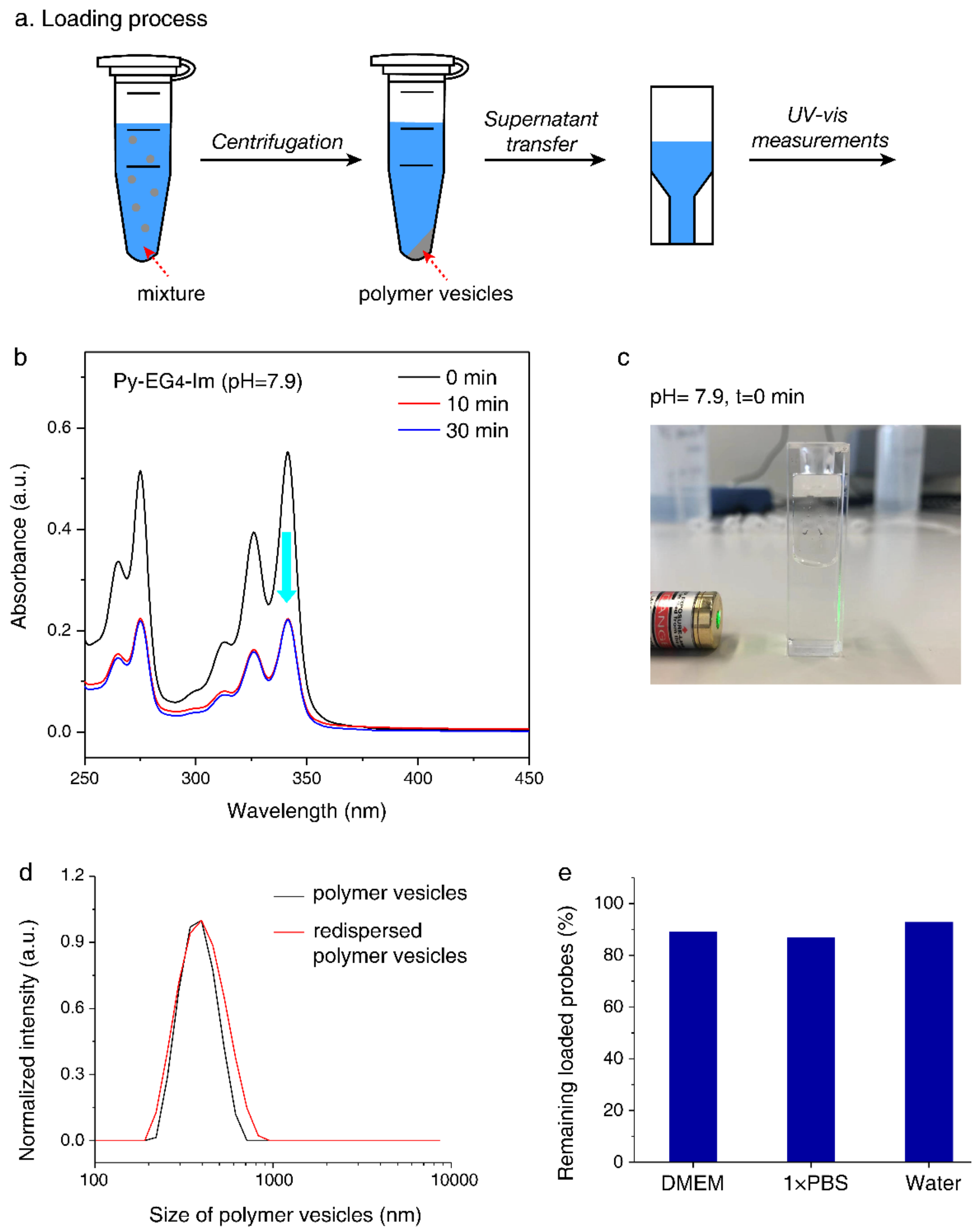

Figure S4. (**a**) The schematic loading process of Py-EG4-Im onto PEG corona of polymer vesicles. (**b**) Typical UV-vis spectra for the supernatant of the loading solution under pH 7.9. Polymer vesicles are removed by centrifugation. (**c**) Optical images of the supernatant obtained at 0 min. The supernatant is illuminated with a laser pointer. (**d**) DLS intensity size distribution of fresh and redispersed polymer vesicles in water. Similar size distribution demonstrates the well re-dispersity of the precipitated vesicles. (**e**) Remaining loaded probes when precipitated polymer vesicles are redispersed in dulbecco's modified Eagle's medium (DMEM), 1×PBS, and water. ~90% of the probes keep their loading state.

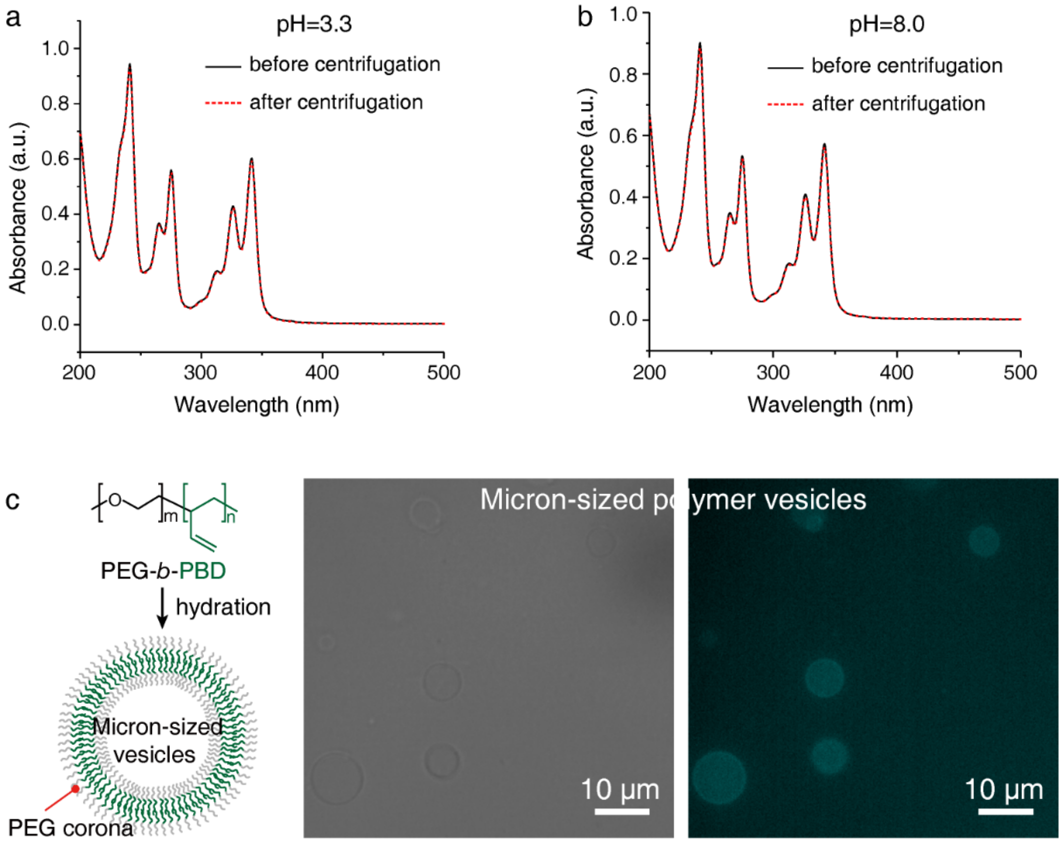

Figure S5. Absorption spectra for the aqueous solution of Py-EG_4-Im (~20 uM) before and after centrifugation under (**a**) pH=3.3 and (**b**) pH=8.0. (**c**) Schematic preparation of micron-sized polymer vesicles with PEG_{22}-*b*-PBD_{37}. Brightfield and fluorescence image of micron-sized polymer vesicles after incubated with Py-EG_4-Im.

To evaluate our loading protocol, the absorption spectra for the aqueous solution of Py-EG_4-Im (~20 uM) before and after centrifugation were measured. As shown in Figure S5a and b, the absorbance of the solution is not affected by centrifugation. This suggested that the decrease in absorbance for loading experiments was not caused by the precipitation of molecular probes during centrifugation.

Moreover, the loading of molecular probes onto polymer vesicles was investigated with a fluorescence microscope. To visualize the loading and distribution of molecular probes on polymer vesicles, we perform the loading experiments with micron-sized polymer vesicles harnessing PEG corona. Micron-sized polymer vesicles were prepared with PEG_{22}-*b*-PBD_{37} by the hydration strategy (Figure S5c). Specifically, PEG_{22}-*b*-PBD_{37} (1.25 mg) dissolved in chloroform was dried with nitrogen stream. After vacuum drying for 12 h, water (3 mL) was

added. The resulting mixture was incubated for 24 h under 60 °C. As-obtained vesicles were incubated with Py-EG_4-Im (33 μM) in a chamber slide. After 10 min, the vesicles were visualized with Leica DMi8 widefield microscope (excitation: 395 nm). The fluorescence of Py is used to visualize the loading and distribution of the molecular probes on vesicles. As shown in Figure S5c, the non-fluorescent polymer vesicles start to emit fluorescence after incubated with Py-EG_4-Im. This confirmed the successful loading of Py-EG_4-Im onto polymer vesicles with PEG corona. The even fluorescence indicated the homogenous distribution of Py-EG_4-Im on the vesicular membrane.

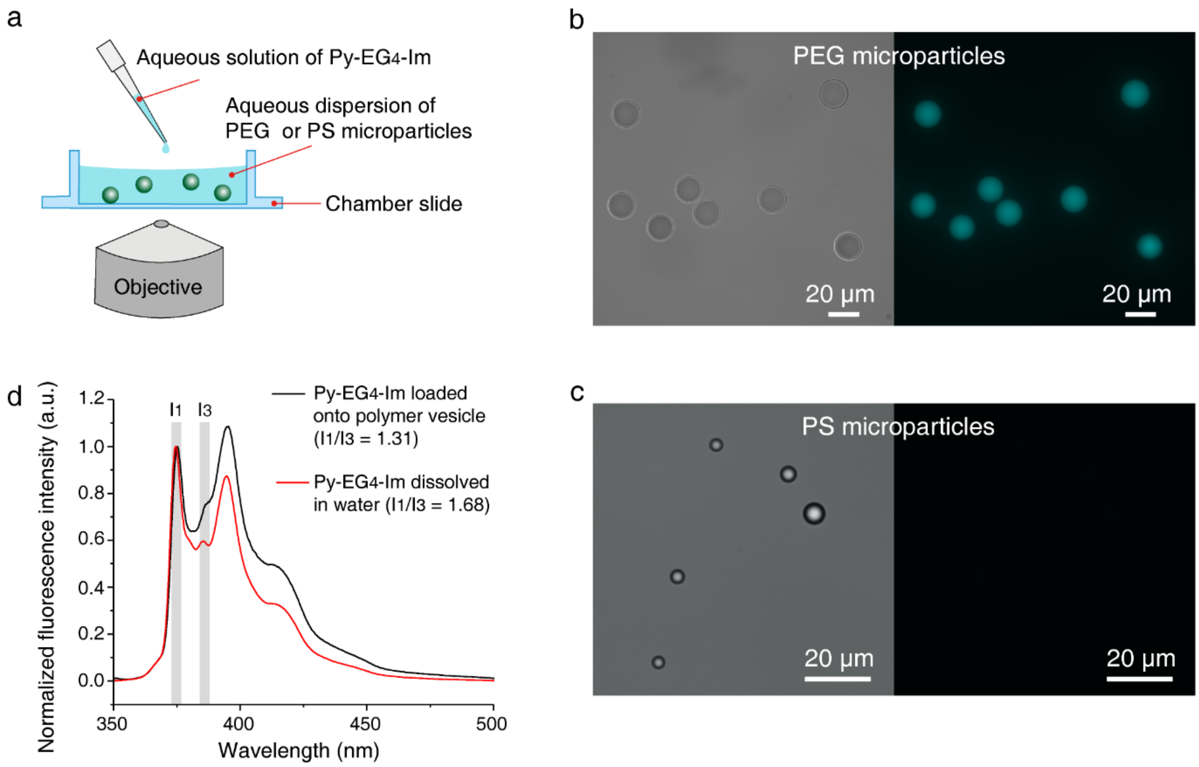

Figure S6. (**a**) Schematic device to investigate the loading of Py-EG4-Im onto PEG and PS microparticles. Brightfield and fluorescence images for the aqueous dispersion of (**b**) PEG microparticles and (**c**) PS microparticles after incubated with Py-EG4-Im (33 μM). All parameters for the microscope are the same for PEG and PS microparticles. (**d**) Normalized fluorescence of Py-EG4-Im loaded onto polymer vesicle and Py-EG4-Im dissolved in water (5 μM). The fluorescence intensity was normalized at the first fluorescence peak (I_1).

To clarify the contribution of the interaction between Py and PS on the loading of molecular probes, we investigate the loading of Py-EG4-Im onto PS and PEG microparticles. Due to the fluorescence of Py, the loading of Py-EG4-Im onto microparticles can be visualized by fluorescence microscope (Figure S6a). Here, PS microparticle is fabricated by interface precipitation as reported previously, which has no surfactants on their surface, allowing us to investigate the interaction between Py-EG4-Im and PS[28]. PEG microparticle is fabricated with a microfluidic setup according to the procedures of our previous work[29].

After incubated with Py-EG4-Im in the chamber slide for 10 min, PEG and PS microparticles were visualized with Leica DMi8 widefield microscope (excitation: 395 nm). PEG microparticles exhibit fluorescence after incubation, while PS microparticles exhibit no fluorescence under the same condition (Figure S6b and S6c). Since both intrinsic PEG and PS

microparticles are not fluorescent, the fluorescence for PEG microparticles is attributed to the loading of Py-EG_4-Im. The strikingly different fluorescence for PEG and PS microparticles indicates that Py is interacting with PEG, while not PS of polymer vesicles.

The position of Py on the vesicular membrane is investigated by measuring its fluorescence. When loaded onto PEG-*b*-PS vesicles, the ratio of the first and third fluorescence peaks (I_1/I_3) of Py-EG_4-Im gets diminished from 1.68 to 1.31 (Figure S6d), suggesting a dramatic decrease of polarity around Py[30]. I_1/I_3 of loaded Py-EG_4-Im is close to the literature value of Py in PEG ($I_1/I_3 = 1.35$)[31] and much higher than Py in PS ($I_1/I_3 = 0.95$)[32]. This further validates that Py is loaded onto the PEG corona while not on the PS core of PEG-*b*-PS vesicles.

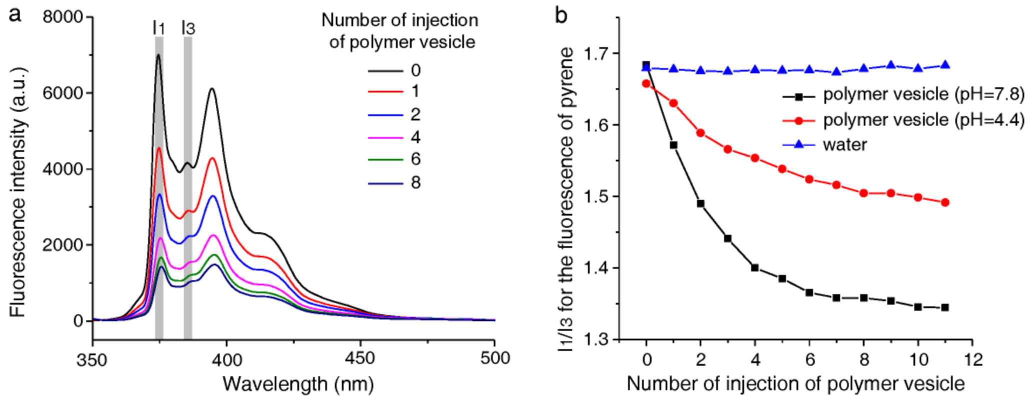

Figure S7. (**a**) The change of fluorescence of Py-EG_4-Im during the injection of polymer vesicle. For each injection, 5 uL dispersion of PEG-*b*-PS vesicle ($6.5\times10^{-9} \pm 8.6\times10^{-10}$ mol L^{-1}) is added into 1 mL Py-EG_4-Im (5 μM) under pH=7.8. (**b**) I_1/I_3 for the fluorescence of Py-EG_4-Im during the injection. The injection of polymer vesicles is conducted under pH=7.8 and pH=4.4. Water is injected as a control.

The loading of Py-EG_4-Im is studied by injecting polymer vesicle into the solution of Py-EG_4-Im. As shown in Figure S7a, the fluorescence of Py-EG_4-Im changed obviously during the injection. Under pH 7.8, I_1/I_3 gradually decreased from 1.68 to 1.36 after six injections of polymer vesicle, while keeping stable during the injection of water (Figure S7b). The approaching of I_1/I_3 to 1.3 indicates that most of Py-EG_4-Im in solution is loaded onto the PEG corona of the polymer vesicle. Under pH 4.4, I_1/I_3 only got decreased to 1.49 after 12 injections, suggesting that quite a lot of Py-EG_4-ImH^+ is still in solution, while not loaded onto polymer vesicles. This is consistent with the poor loading of Py-EG_4-Im under acidic conditions.

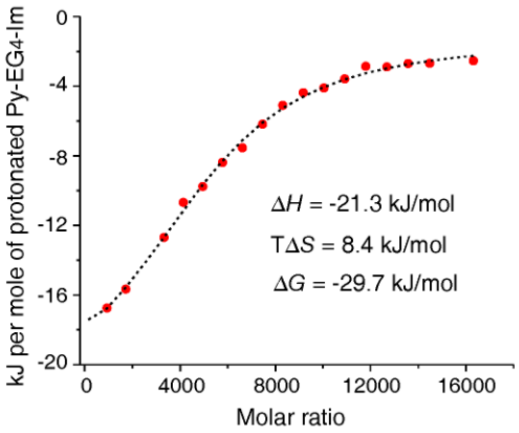

Figure S8. ITC analysis for the binding of Py-EG_4-ImH^+ and PEG corona. The fitted plot (dotted line) is produced with ITC Analysis Software (MicroCal PEAQ). The concentration of polymer vesicles is 3.84×10^{-9} M. The concentration of Py-EG_4-ImH^+ is 302 μM. The change of Gibbs free energy (ΔG), enthalpy (ΔH), and entropy ($T\Delta S$) are -29.7 kJ/mol, -21.3 kJ/mol, and 8.4 kJ/mol. The negative ΔG suggests the thermodynamically favorable binding of Py-EG_4-ImH^+ and PEG corona.

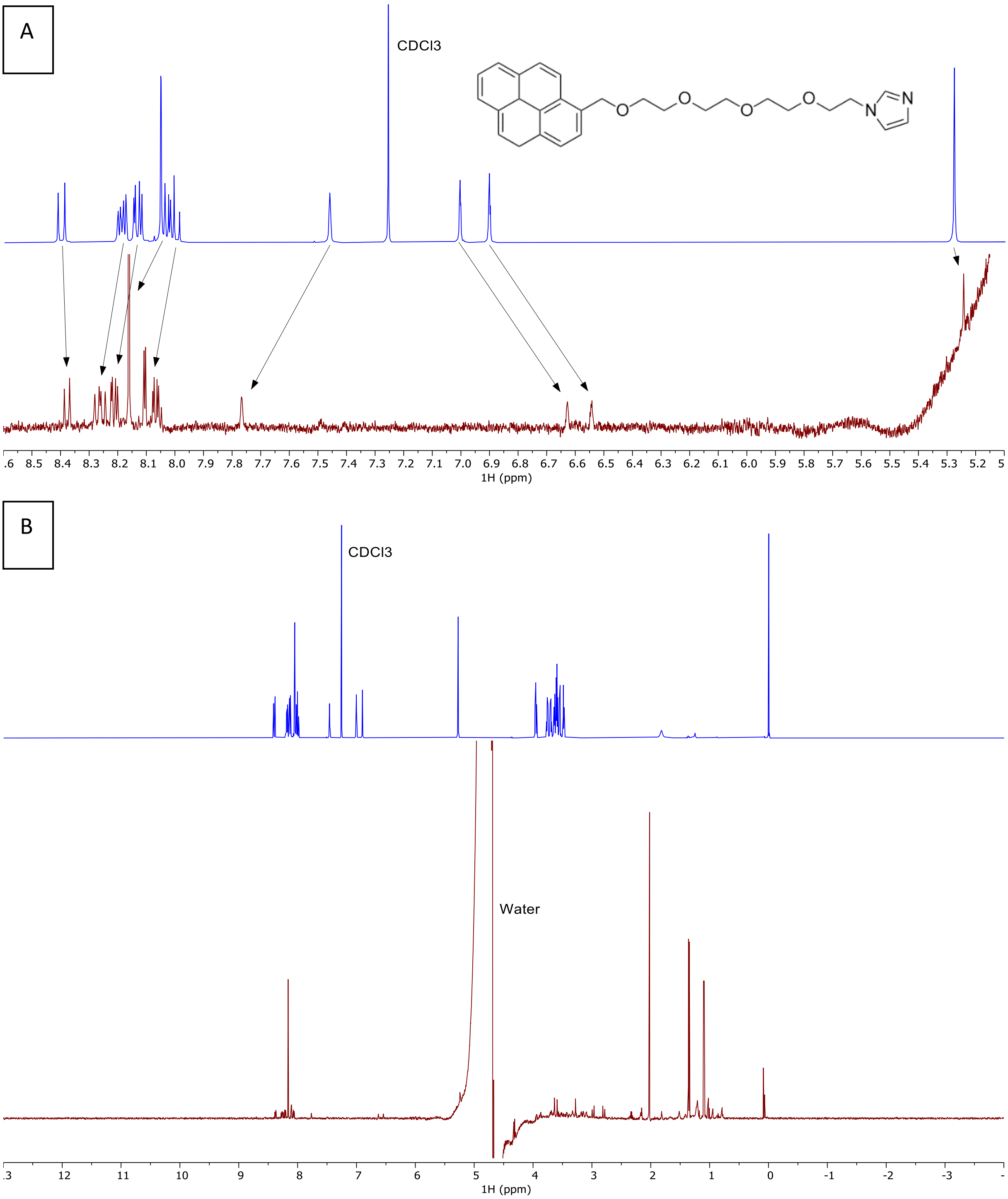

Figure S9. Overlay of ^{1}H NMR spectra zoomed-in (A) and full (B) of the as-synthesized Py-EG4-Im (blue line) and unloaded Py-EG4-Im (red line). Top: Py-EG4-Im dissolved in $CDCl_3$ (NS=16). Bottom: unloaded Py-EG4-Im in D_2O by adding H_2SO_4 (NS=4096, solvent suppressed). The unloaded probe is evident by the peaks of the pyrene aromatics (8.5–7.9 ppm) and imidazole (7.8–6.5 ppm). The peaks are slightly shifted due to the change in solvent ($CDCl_3$ to D_2O), as shown by the arrows. The incline from 5.4 ppm is due to the water peak.

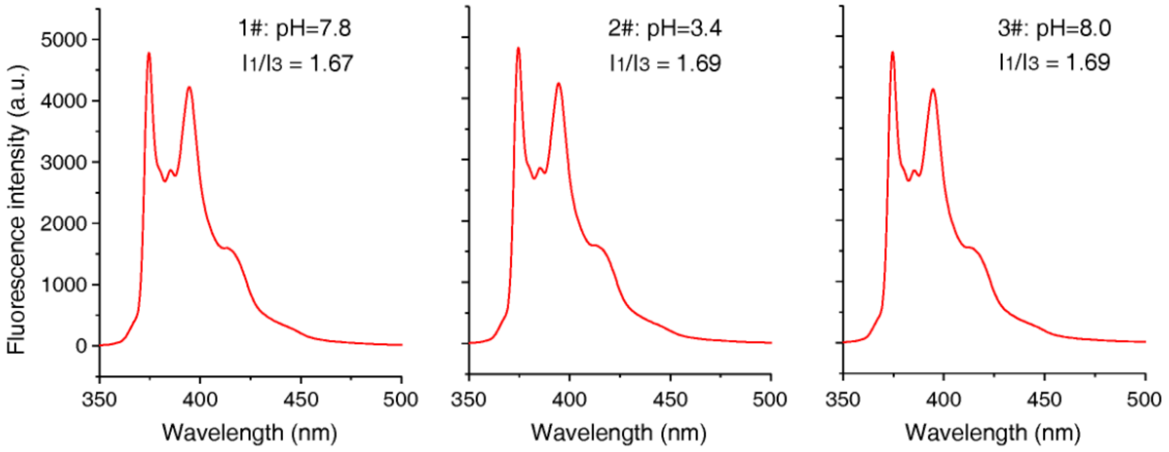

Figure S10. Fluorescence for the aqueous solution of Py-EG$_4$-Im (20 µM) when pH is changed between 7.8-8.0 and 3.4. The fluorescence spectrum and I_1/I_3 (1.67-1.69) for Py-EG$_4$-Im is not affected by the solution pH. This confirms that Py-EG$_4$-Im keeps the soluble state, while not forming aggregates when pH is changed between 7.8-8.0 and 3.4.

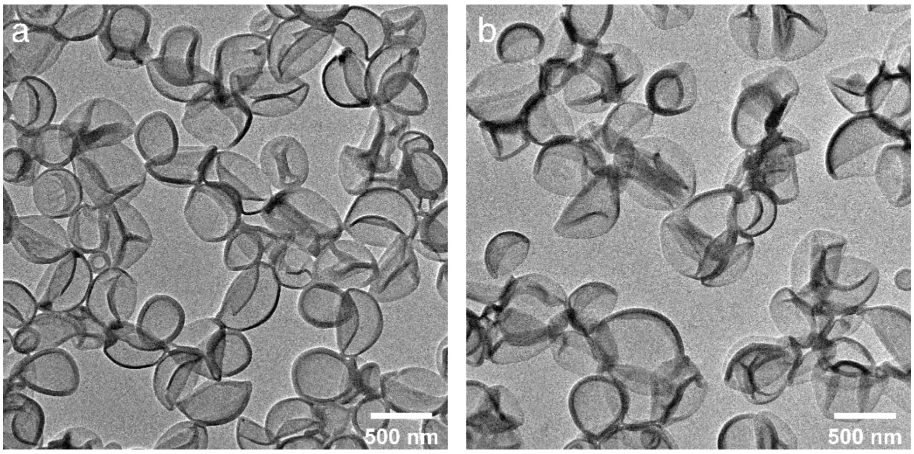

Figure S11. TEM image of polymer vesicles (**a**) before, and (**b**) after loading/unloading cycles. Polymer vesicles keep intact during the experiment, which is attributed to the glassy PS core.

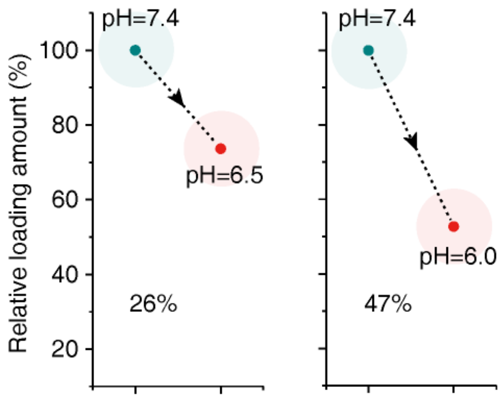

Figure S12. The unloading of molecular probes when pH is decreased from 7.4 to 6.5 and 6.0. 7.4 represents physiological pH, 6.5 represents the pH in early endosome, and 6.0 represents the pH in late endosome. 26% and 47% of the loaded Py-EG_4-Im are unloaded when pH is decreased from 7.4 to 6.5 and 6.0, respectively.

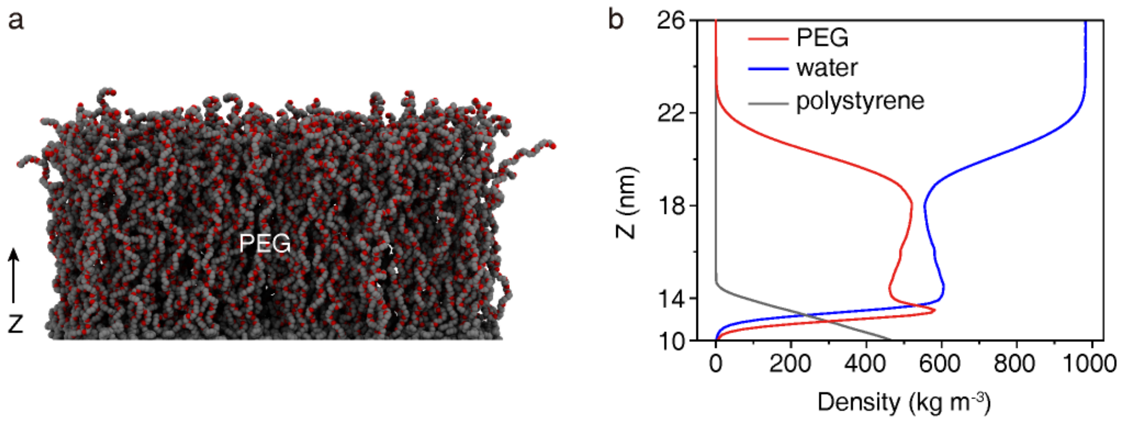

Figure S13. (**a**) A representative snapshot of PEG corona in the space-filling model: dark grey C; red O. (**b**) The density of PEG, water, and polystyrene along Z-direction for (a). The density plot is averaged over the entire simulation (0-100 ns).

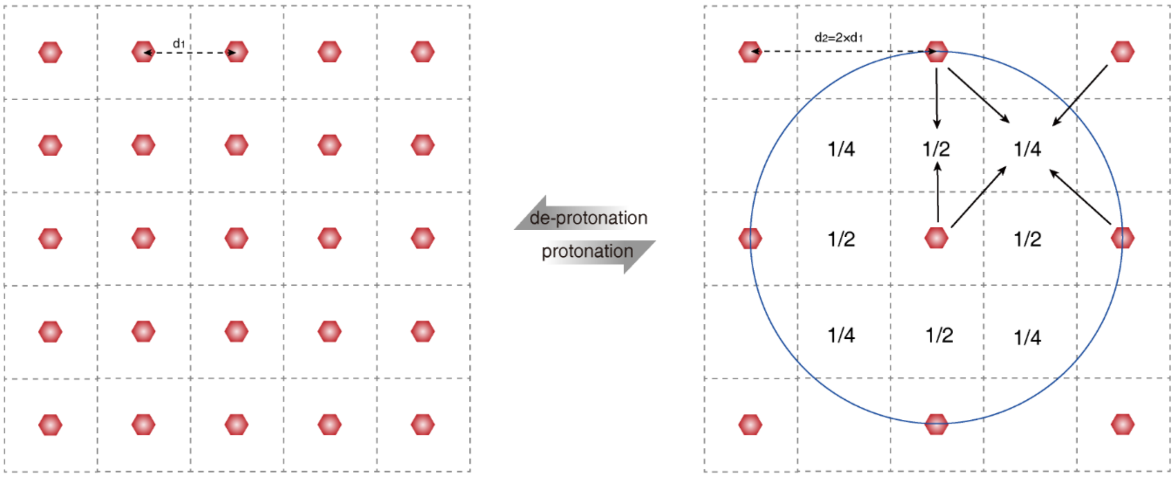

Figure S14. Schematic distribution of Py-EG4-Im on the surface of PEG corona during the loading and unloading cycle. The red hexagon represents the loaded -Im. By assuming a 2-fold increase over the distance between the loaded probes during unloading, each probe would result in 3 neighboring probes being released into the solution by electrostatic repulsion. This corresponds to an unloading amount of 75%, which is close to our experimental results.

By assuming the homogenous distribution of probes on the vesicular membrane, the distance (d) between loaded molecular probes can be calculated with equation *S1*:

$$d = \sqrt{\frac{n4\pi r^2}{N}} \qquad (S1)$$

where N is the loading amount of probes, n is the number of polymer vesicles, and r is the radius of polymer vesicles. In multiple cycling experiments, n is 2.07×10^{-7} μmol as measured by Nanosight, and r is 229 nm as measured by DLS. With N obtained from the UV-vis spectrometer, d can be calculated.

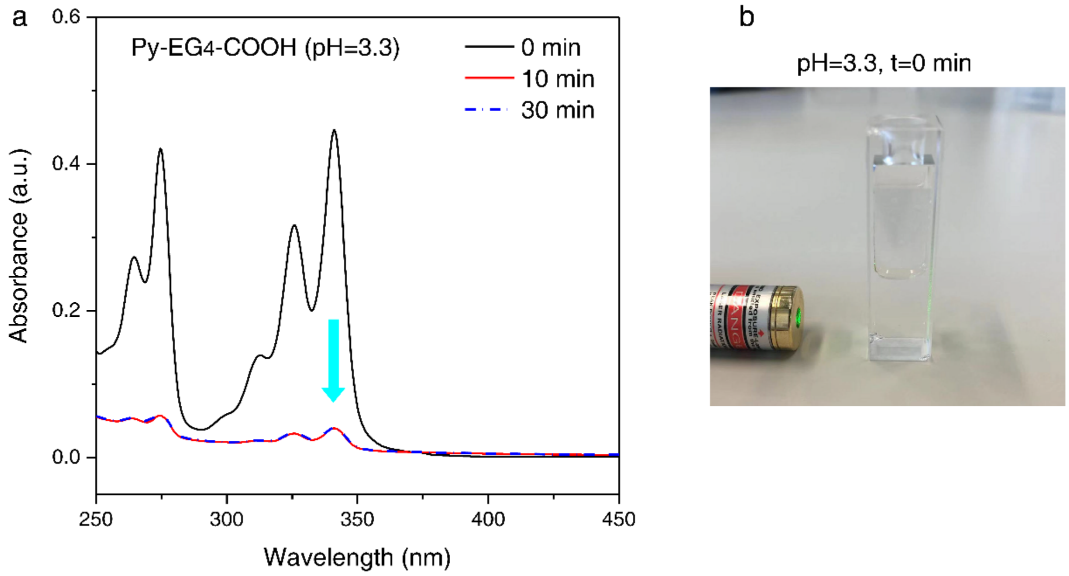

Figure S15. The loading of Py-EG4-COOH onto polymer vesicles. (**a**) Typical UV-vis spectra for the supernatant of the loading solution under pH 3.3. (**b**) Optical image of the supernatant obtained at 0 min. The supernatant is illuminated with a laser pointer.

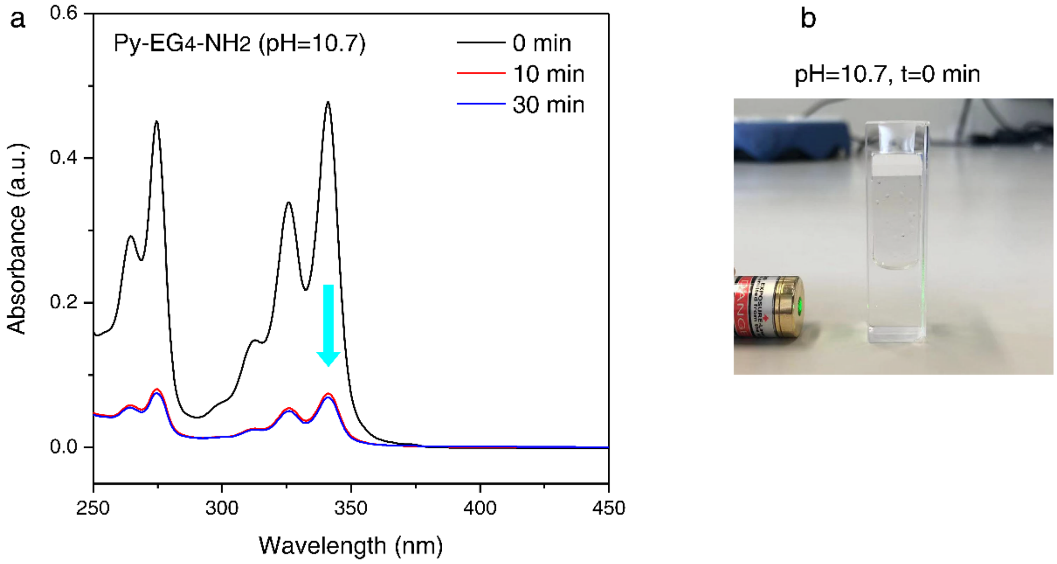

Figure S16. The loading of Py-EG$_4$-NH$_2$ onto polymer vesicles. (**a**) Typical UV-vis spectra for the supernatant of the loading solution under pH 10.7. (**b**) Optical image of the supernatant obtained at 0 min. The supernatant is illuminated with a laser pointer.

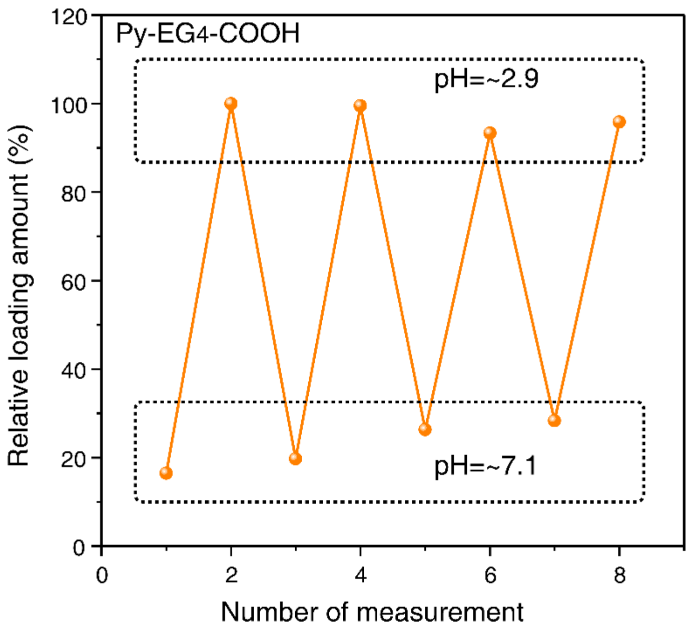

Figure S17. The reversible loading and unloading of Py-EG_4-COOH onto the PEG corona of polymer vesicles by switching the pH between 7.1 and 2.9.

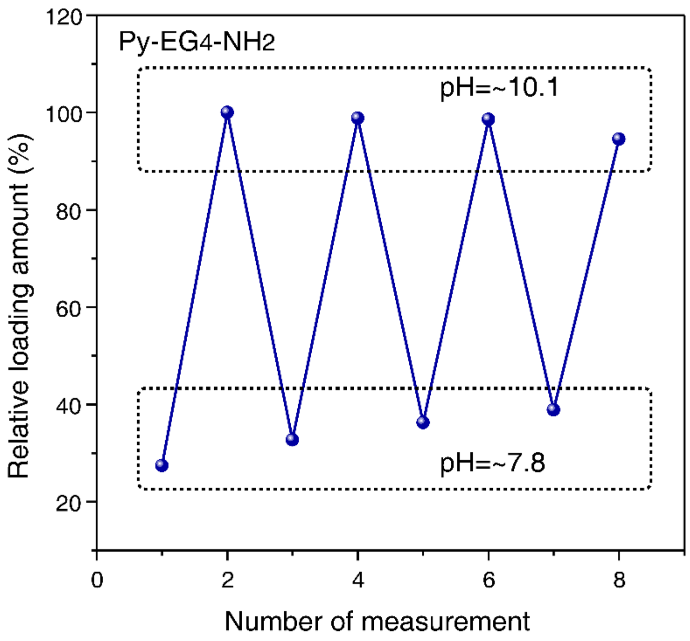

Figure S18. The reversible loading and unloading of Py-EG_4-NH_2 onto the PEG corona of polymer vesicles by switching the pH between 7.8 and 10.1.

7. Supplementary References:

1. Zhang, S.; Li, W.; Luan, J.; Srivastava, A.; Carnevale, V.; Klein, M. L.; Sun, J.; Wang, D.; Teora, S. P.; Rijpkema, S. J.; Meeldijk, J. D.; Wilson, D. A. Adaptive insertion of a hydrophobic anchor into a poly(ethylene glycol) host for programmable surface functionalization. *Nat. Chem.* **2023**, *15*, 240-247.
2. Martinez, L.; Andrade, R.; Birgin, E. G.; Martinez, J. M. PACKMOL: a package for building initial configurations for molecular dynamics simulations. *J. Comput. Chem.* **2009**, *30*, 2157-2164.
3. Vermaas, J. V.; Hardy, D. J.; Stone, J. E.; Tajkhorshid, E.; Kohlmeyer, A. TopoGromacs: Automated topology conversion from CHARMM to GROMACS within VMD. *J. Chem. Inf. Model.* **2016**, *56*, 1112-1116.
4. Humphrey, W.; Dalke, A.; Schulten, K. VMD: Visual molecular dynamics. *J. Mol. Graph.* **1996**, *14*, 33-38.
5. Jo, S.; Kim, T.; Iyer, V. G.; Im, W. CHARMM-GUI: a web-based graphical user interface for CHARMM. *J. Comput. Chem.* **2008**, *29*, 1859-1865.
6. Choi, Y. K.; Park, S. J.; Park, S.; Kim, S.; Kern, N. R.; Lee, J.; Im, W. CHARMM-GUI polymer builder for modeling and simulation of synthetic polymers. *J. Chem. Theory Comput.* **2021**, *17*, 2431-2443.
7. Vanommeslaeghe, K.; Hatcher, E.; Acharya, C.; Kundu, S.; Zhong, S.; Shim, J.; Darian, E.; Guvench, O.; Lopes, P.; Vorobyov, I.; Mackerell Jr., A. D. CHARMM general force field: A force field for drug-like molecules compatible with the CHARMM all-atom additive biological force fields. *J. Comput. Chem.* **2010**, *31*, 671-690.
8. Yu, W.; He, X.; Vanommeslaeghe, K.; MacKerell Jr., A. D. Extension of the CHARMM general force field to sulfonyl-containing compounds and its utility in biomolecular simulations. *J. Comput. Chem.* **2012**, *33*, 2451-2468.
9. Vanommeslaeghe, K.; MacKerell Jr., A. D. Automation of the CHARMM General Force Field (CGenFF) I: bond perception and atom typing. *J. Chem. Inf. Model.* 2012, *52*, 3144-3154.
10. Vanommeslaeghe, K.; Raman, E. P.; Mackerell Jr., A. D. Automation of the CHARMM General Force Field (CGenFF) II: assignment of bonded parameters and partial atomic charges. *J. Chem. Inf. Model.* **2012**, *52*, 3155-3168.

11. Klauda, J. B.; Venable, R. M.; Freites, J. A.; O'Connor, J. W.; Tobias, D. J.; Mondragon-Ramirez, C.; Vorobyov, I.; MacKerell, Jr., A. D.; Pastor, R. W. Update of the CHARMM all-atom additive force field for lipids: validation on six lipid types. *J. Phys. Chem. B* **2010**, *114*, 7830-7843.
12. Bussi, G.; Donadio, D.; Parrinello, M. Canonical sampling through velocity rescaling. *J. Chem. Phys.* **2007**, *126*, 014101.
13. Berendsen, H. J. C.; Postma, J. P. M.; van Gunsteren, W. F.; DiNola, A.; Haak, J. R. Molecular dynamics with coupling to an external bath. *J. Chem. Phys.* **1984**, *81*, 3684-3690.
14. Essmann, U.; Perera, L.; Berkowitz, M. L.; Darden, T.; Lee, H.; Pedersen, L. G. A smooth particle mesh Ewald method. *J. Chem. Phys.* **1995**, *103*, 8577.
15. Allen, T. D.; Allen, M. P. Oxford University Press, New York, **1987**.
16. Darden, T.; York, D.; Pedersen, L. Particle mesh Ewald: An N · log (N) method for Ewald sums in large systems. *J. Chem. Phys.* **1993**, *98*, 10089-10092.
17. Hess, B.; Bekker, H.; Berendsen, H. J. C.; Fraaije, J. G. E. M. LINCS: a linear constraint solver for molecular simulations. *Journal of computational chemistry. J. Comput. Chem.* **1997**, *18*, 1463-1472.
18. Mark, P.; Nilsson, L. Structure and dynamics of the TIP3P, SPC, and SPC/E water models at 298 K. *J. Phys. Chem. A* **2001**, *105*, 9954-9960.
19. Jorgensen, W. L.; Chandrasekhar, J.; Madura, J. D.; Impey, R. W.; Klein, M. L. Comparison of simple potential functions for simulating liquid water. *J. Chem. Phys.* **1983**, *79*, 926-935.
20. Berendsen, H. J. C.; Dijkstra, E. J.; Achterop, S.; Vondrumen, R.; Vanderspoel, D.; Sijbers, A.; Keegstra, H.; Renardus, M. K. R. Gromacs-a parallel computer for molecular-dynamics simulations. *Physics computing* **1993**, 252-256.
21. Berendsen, H. J. C.; van der Spoel, D.; van Drunen, R. GROMACS: A message-passing parallel molecular dynamics implementation. *Comput. Phys. Commun.* **1995**, *91*, 43-56.
22. Lindahl, E.; Hess, B.; van der Spoel, D. GROMACS 3.0: a package for molecular simulation and trajectory analysis. *Molecular modeling annual* **2001**, *7*, 306-317.
23. van der Spoel, D.; Lindahl, E.; Hess, B.; Groenhof, G.; Mark, A. E.; Berendsen, H. J. ROMACS: fast, flexible, and free. *J. Comput. Chem.* **2005**, *26*, 1701-1718.

24. Hess, B.; Kutzner, C.; van der Spoel, D.; Lindahl, E. GROMACS 4: algorithms for highly efficient, load-balanced, and scalable molecular simulation. *J. Chem. Theory Comput.* **2008**, *4*, 435-447.
25. Pronk, S.; Páll, S.; Schulz, R.; Larsson, P.; Bjelkmar, P.; Apostolov, R.; Shirts, M. R.; Smith, J. C.; Kasson, P. M.; van der Spoel, D.; Hess, B.; Lindahl, E. GROMACS 4.5: a high-throughput and highly parallel open source molecular simulation toolkit. *Bioinformatics* **2013**, *29*, 845-854.
26. Abraham, M. J.; Murtola, T.; Schulz, R.; Páll, S.; Smith, J. C.; Hess, B.; Lindahl, E. GROMACS: High performance molecular simulations through multi-level parallelism from laptops to supercomputers. *SoftwareX* **2015**, *1*, 19-25.
27. Buwalda, S.; Al Samad, A.; El Jundi, A.; Bethry, A.; Bakkour, Y.; Coudane, J.; Nottelet, B. Stabilization of poly (ethylene glycol)-poly (ε-caprolactone) star block copolymer micelles via aromatic groups for improved drug delivery properties. *J. Colloid Interface Sci.* **2018**, *514*, 468-478.
28. Yamamoto, Y.; Okada, D.; Kushida, S.; Ngara, Z. S.; Oki, O. Fabrication of polymer microspheres for optical resonator and laser applications. *J. Vis. Exp.* **2017**, *124*, e55934.
29. Keller, S.; Teora, S. P.; Hu, G. X.; Nijemeisland, M.; Wilson, D. A. High-throughput design of biocompatible enzyme-based hydrogel microparticles with autonomous movement. *Angew. Chem. Int. Ed.* **2018**, *57*, 9814-9817.
30. Kalyanasundaram, K.; Thomas, J. K. Solvent-Dependent Fluorescence of Pyrene-3-carboxaldehyde and its applications in the estimation of polarity at micelle-water interfaces. *J. Phys. Chem.* **1977**, *81*, 2176-2180.
31. Hoang, K. C. Aqueous solubilization of highly fluorinated molecules by semifluorinated surfactants. *Langmuir* **2004**, *20*, 7347-7350.
32. Gu, S.; Anzai, N.; Nagao, D.; Kobayashi, Y.; Konno, M. Preparation of fluorescent polymer particles by emulsion polymerization. *e-Polymers* **2005**, *064*.

8. Characterization of molecular probes

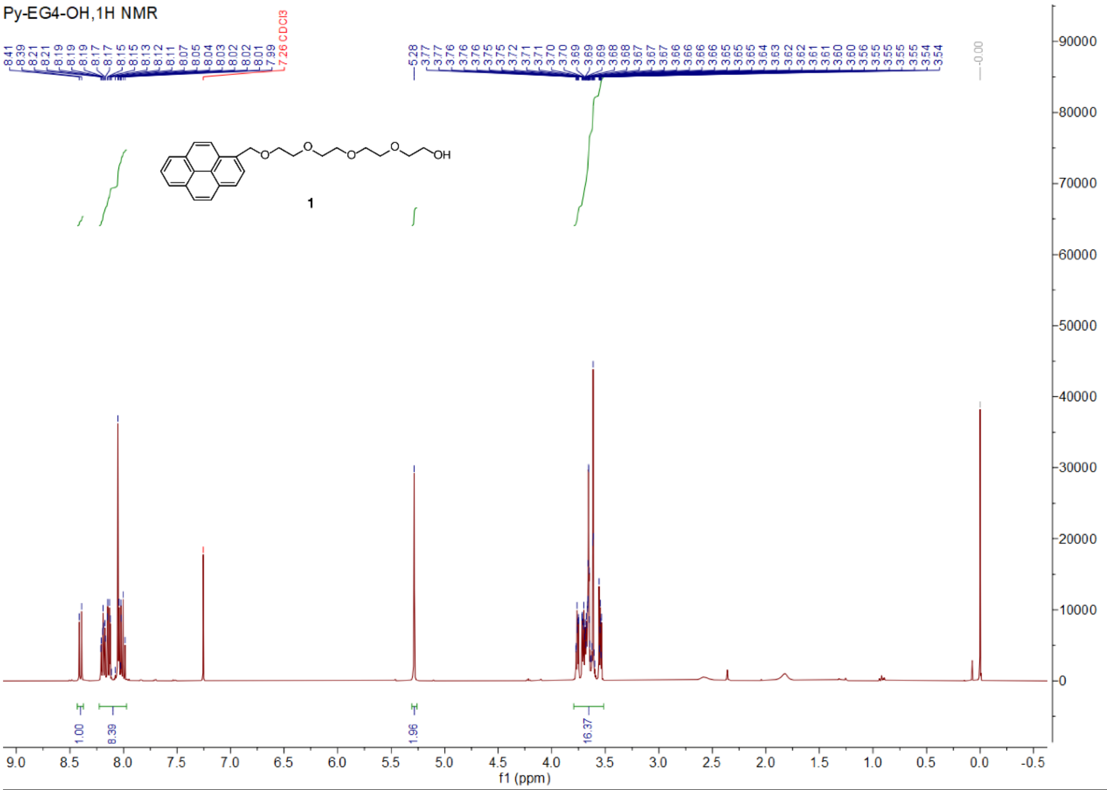

^{1}H NMR of Py-EG$_4$-OH **1** in $CDCl_3$.

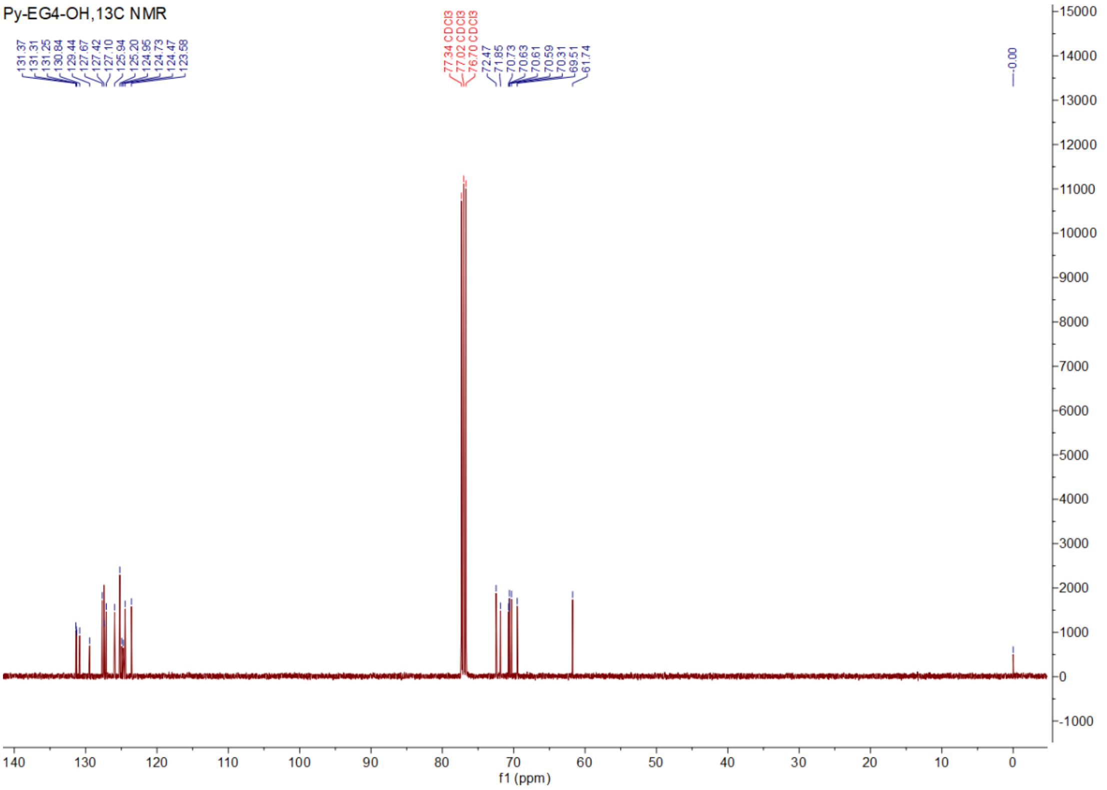

^{13}C NMR of Py-EG$_4$-OH **1** in $CDCl_3$.

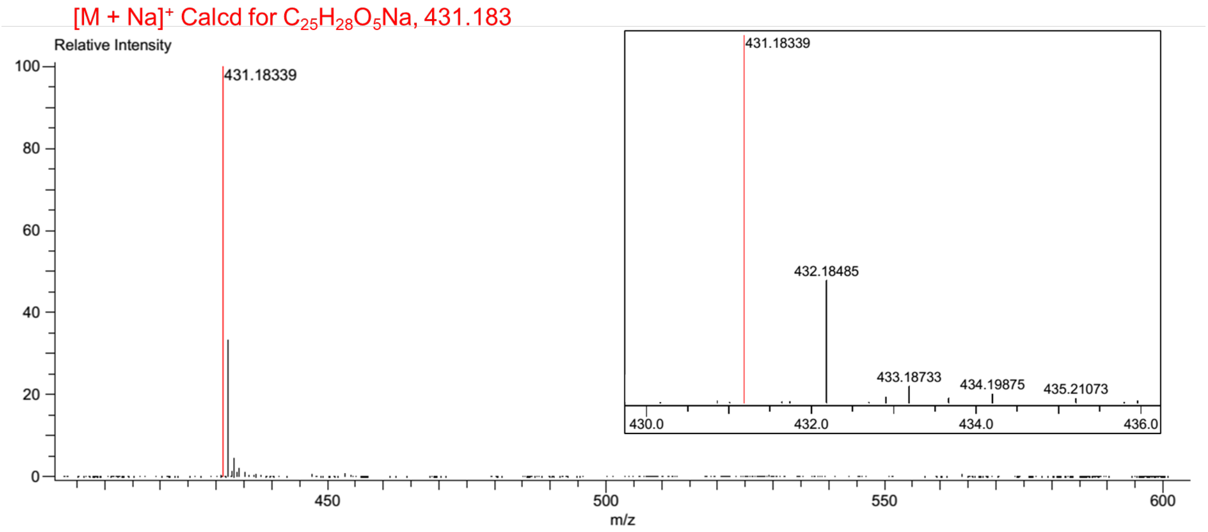

HR-MS of Py-EG$_4$-OH **1**.

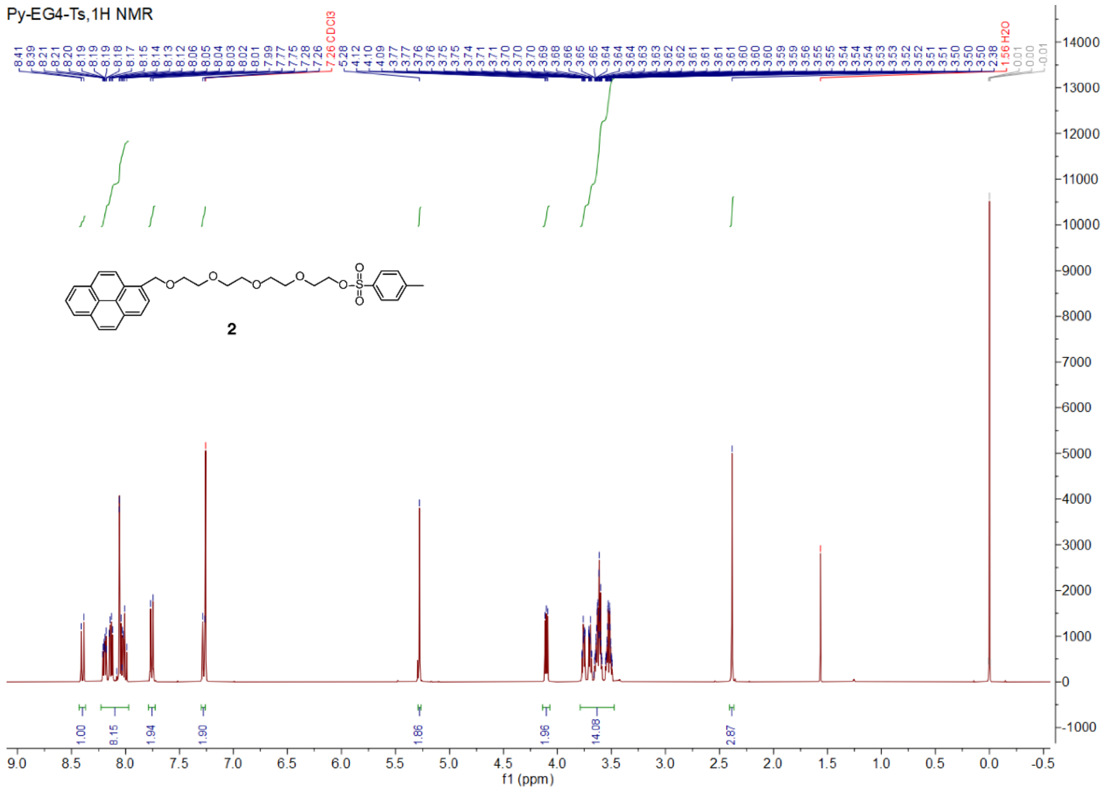

^{1}H NMR of Py-EG$_4$-Ts **2** in $CDCl_3$.

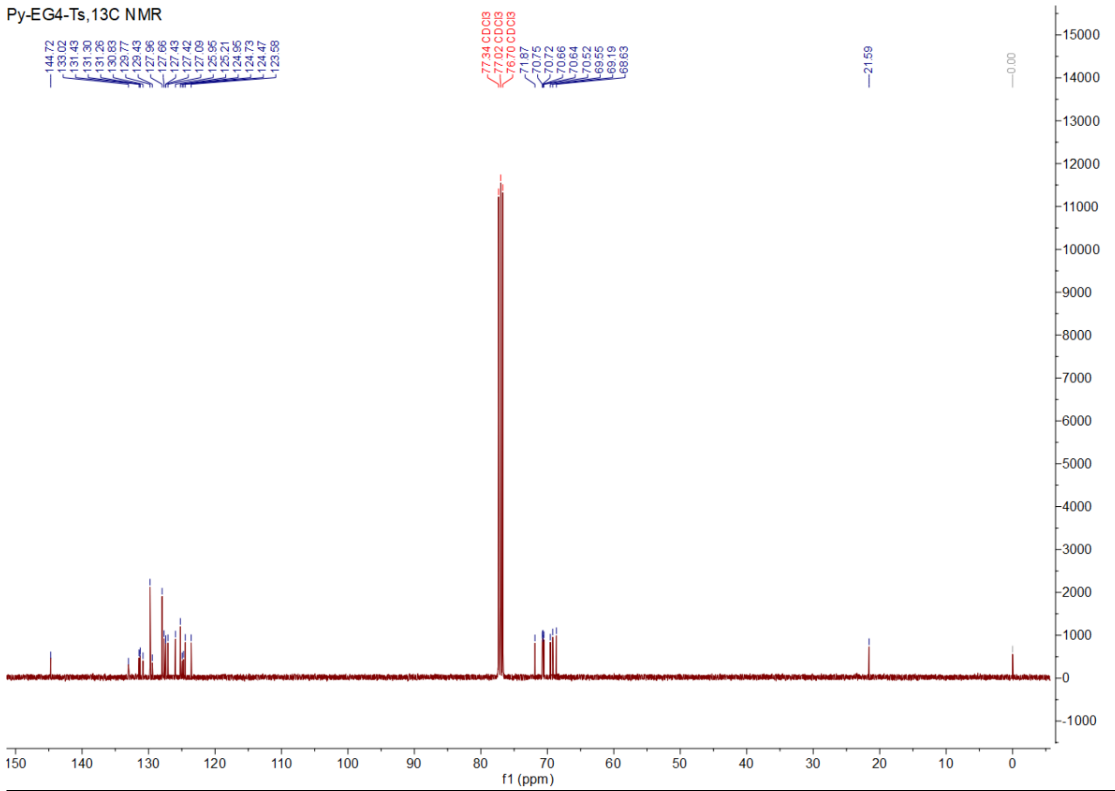

^{13}C NMR of Py-EG$_4$-Ts **2** in $CDCl_3$.

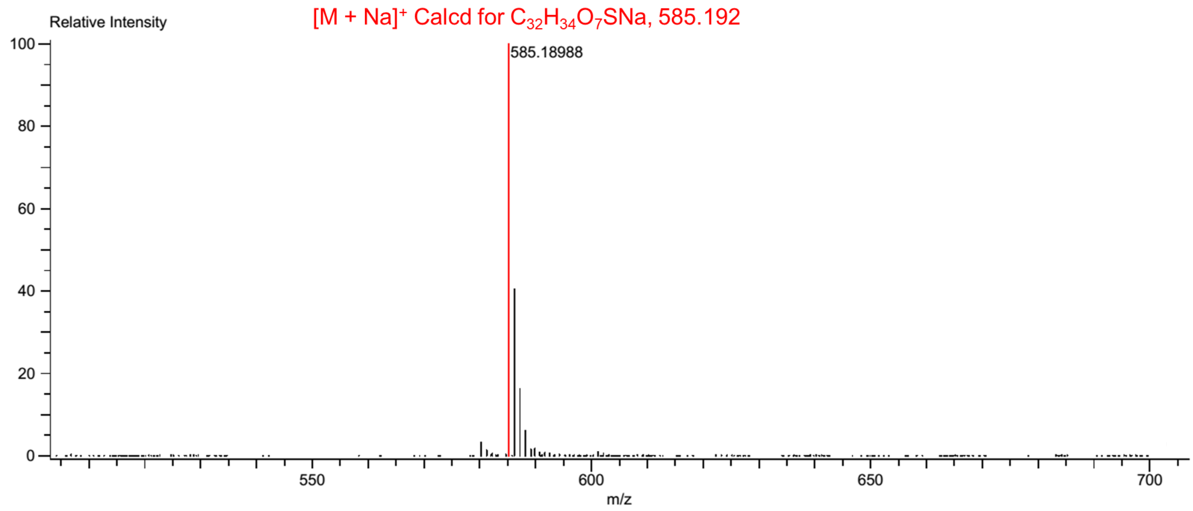

HR-MS of Py-EG4-Ts **2**.

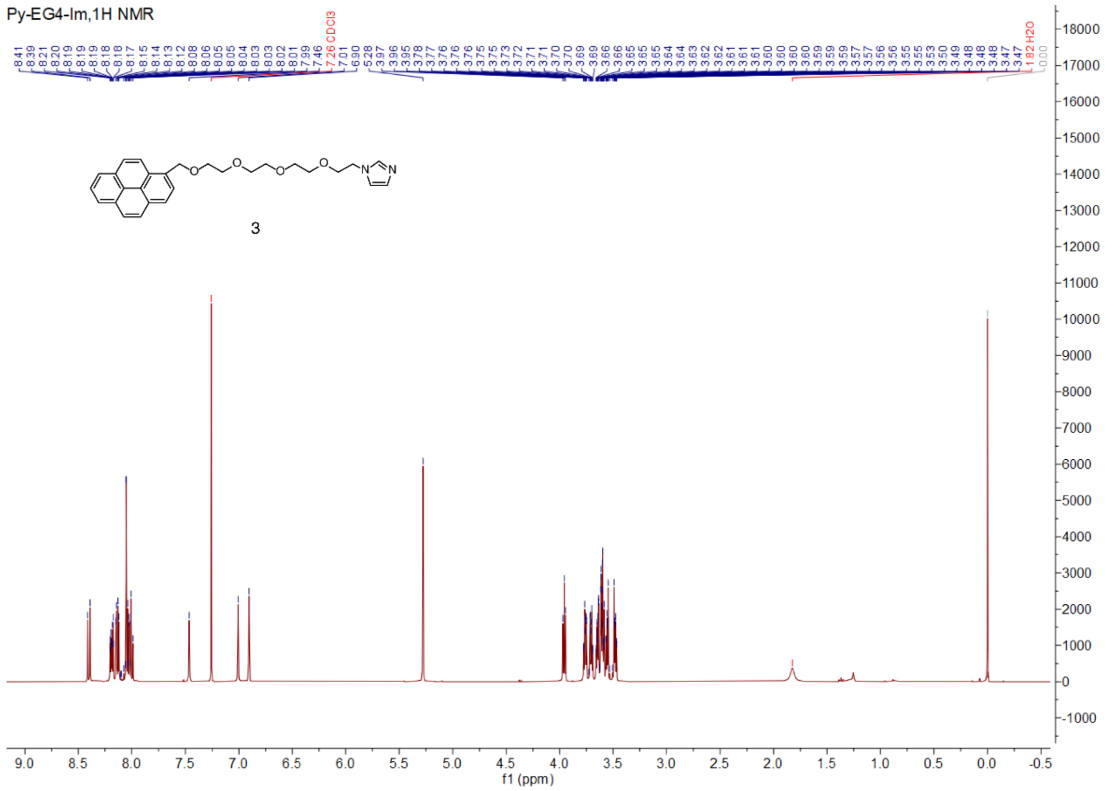

^{1}H NMR of Py-EG$_4$-Im **3** in $CDCl_3$.

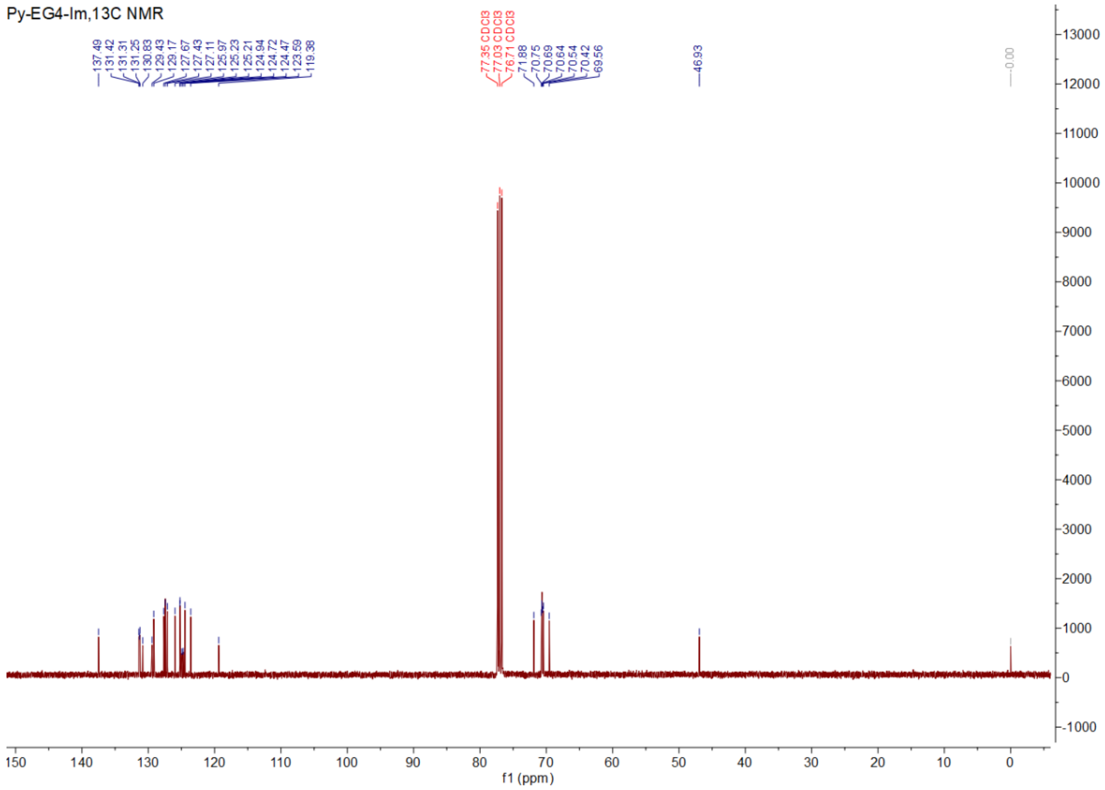

^{13}C NMR of Py-EG$_4$-Im **3** in $CDCl_3$.

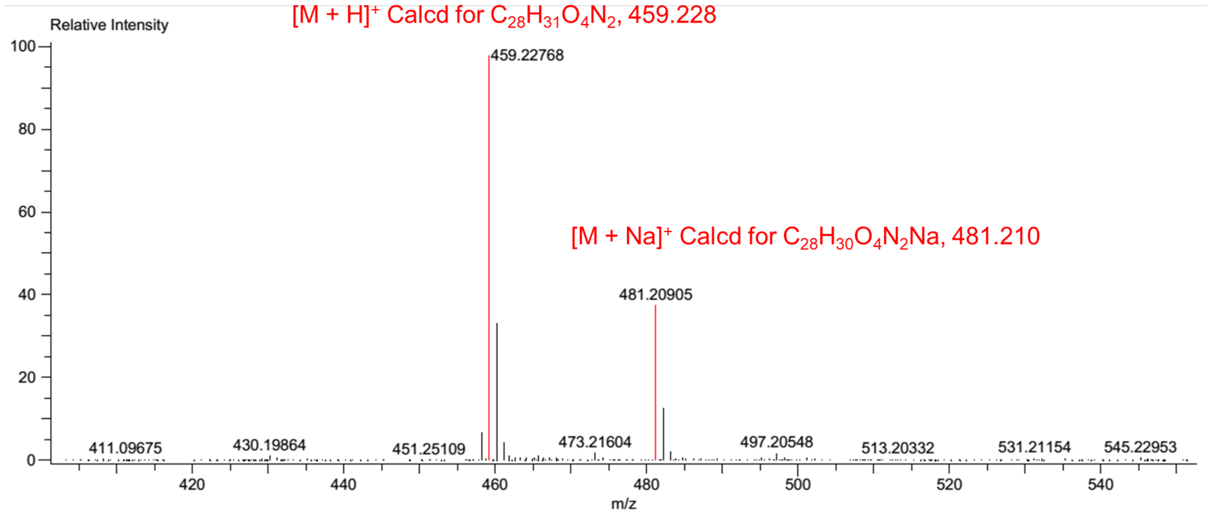

HR-MS of Py-EG$_4$-Im **3**.

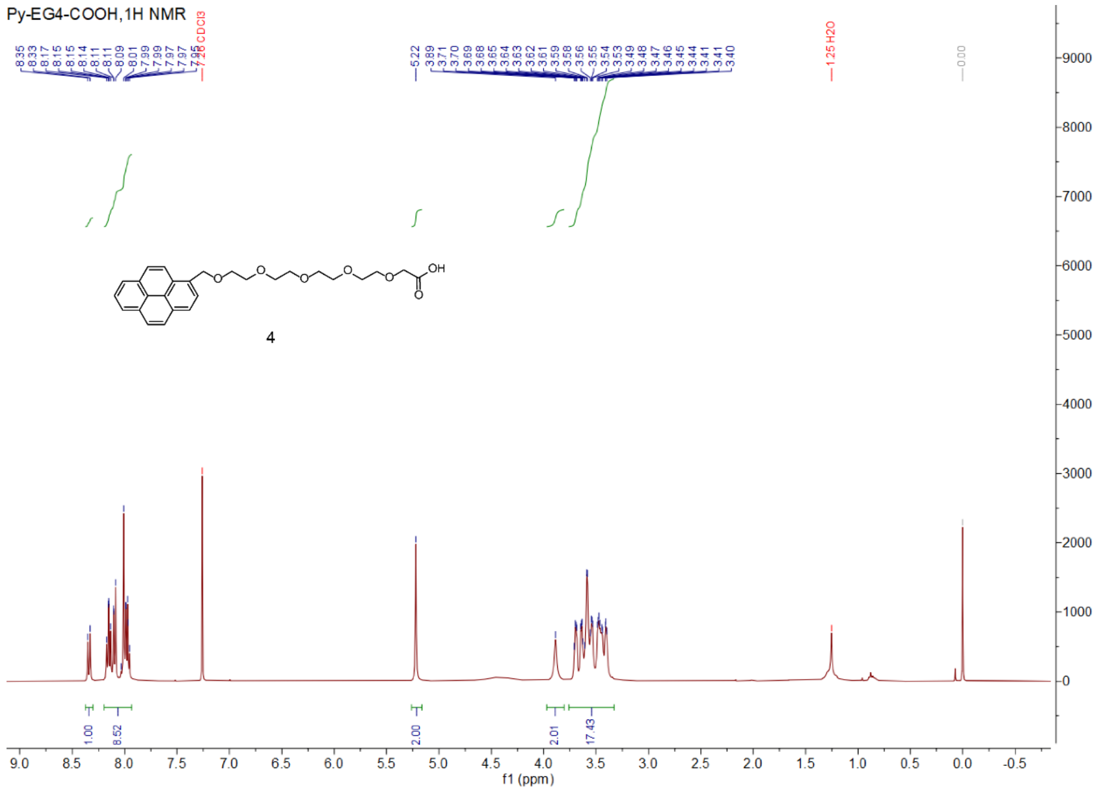

^{1}H NMR of Py-EG_4-COOH **4** in $CDCl_3$.

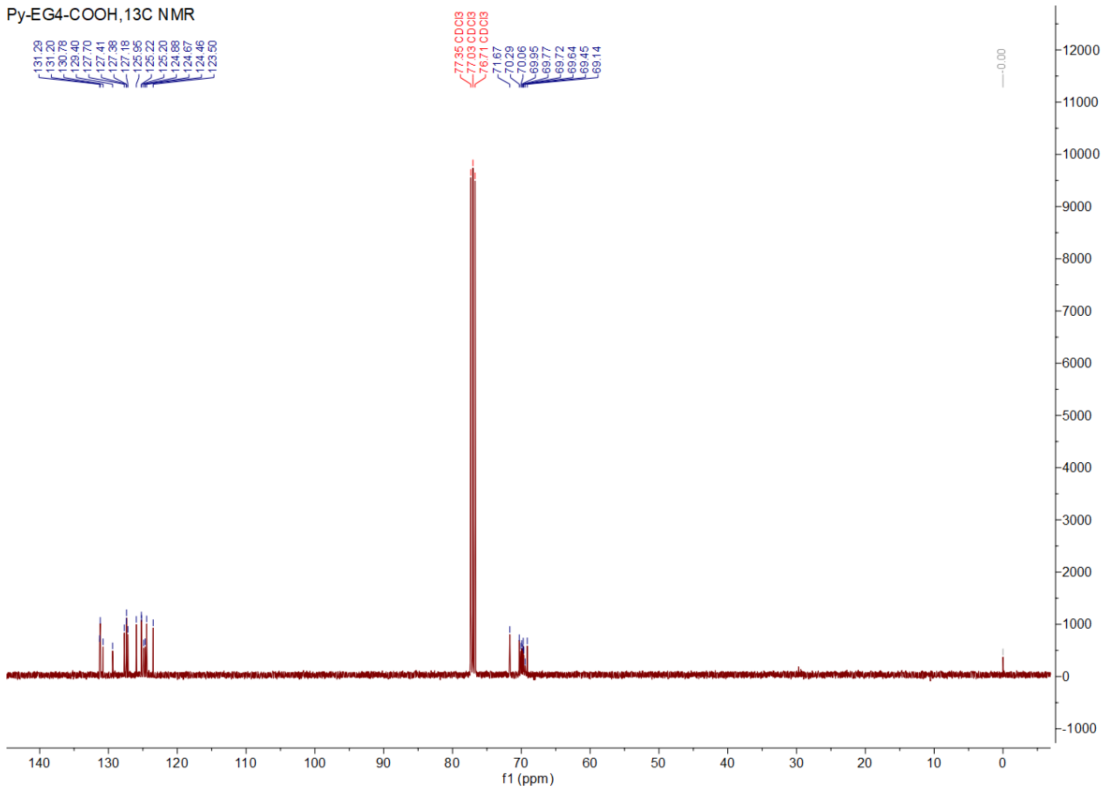

^{13}C NMR of Py-EG_4-COOH **4** in $CDCl_3$.

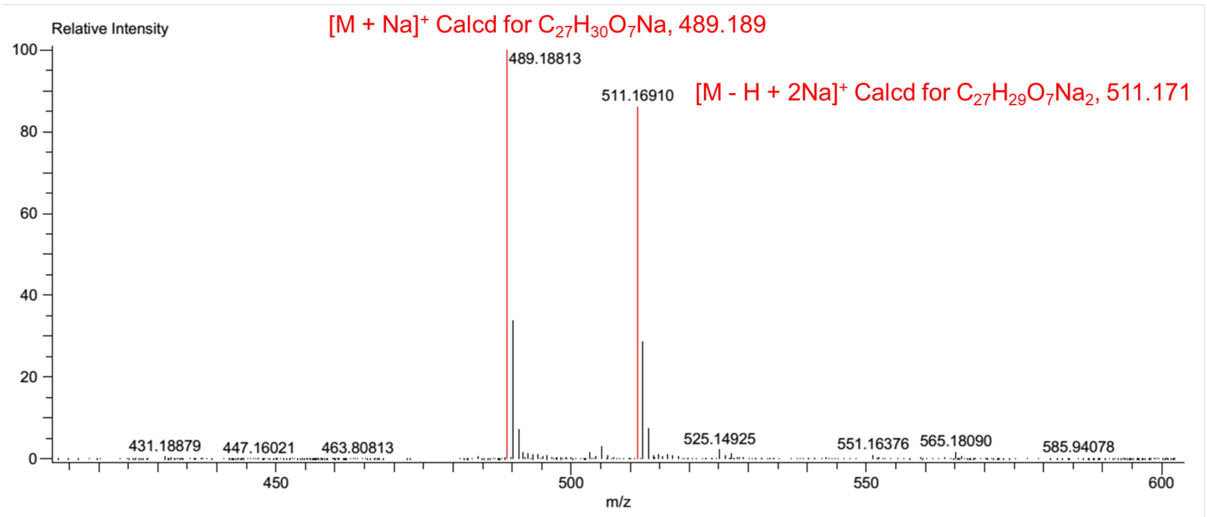

HR-MS of Py-EG_4-COOH **4**.

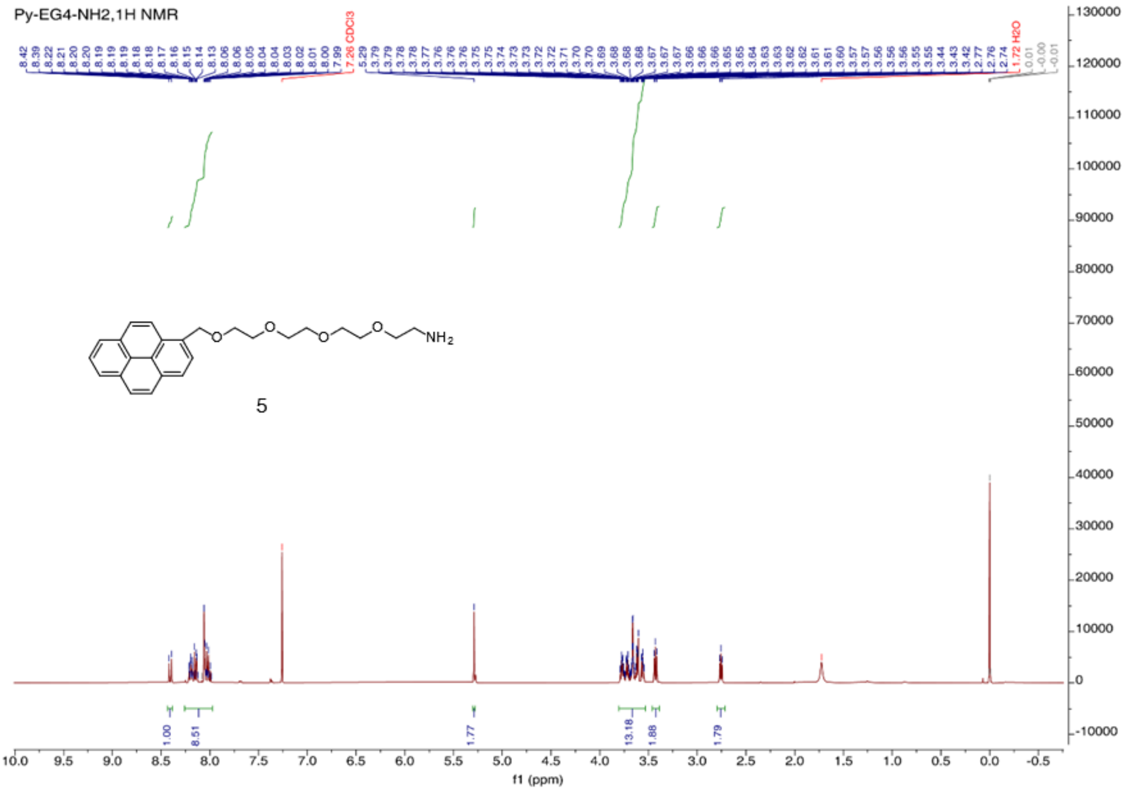

^{1}H NMR of Py-EG$_4$-NH$_2$ **5** in CDCl$_3$

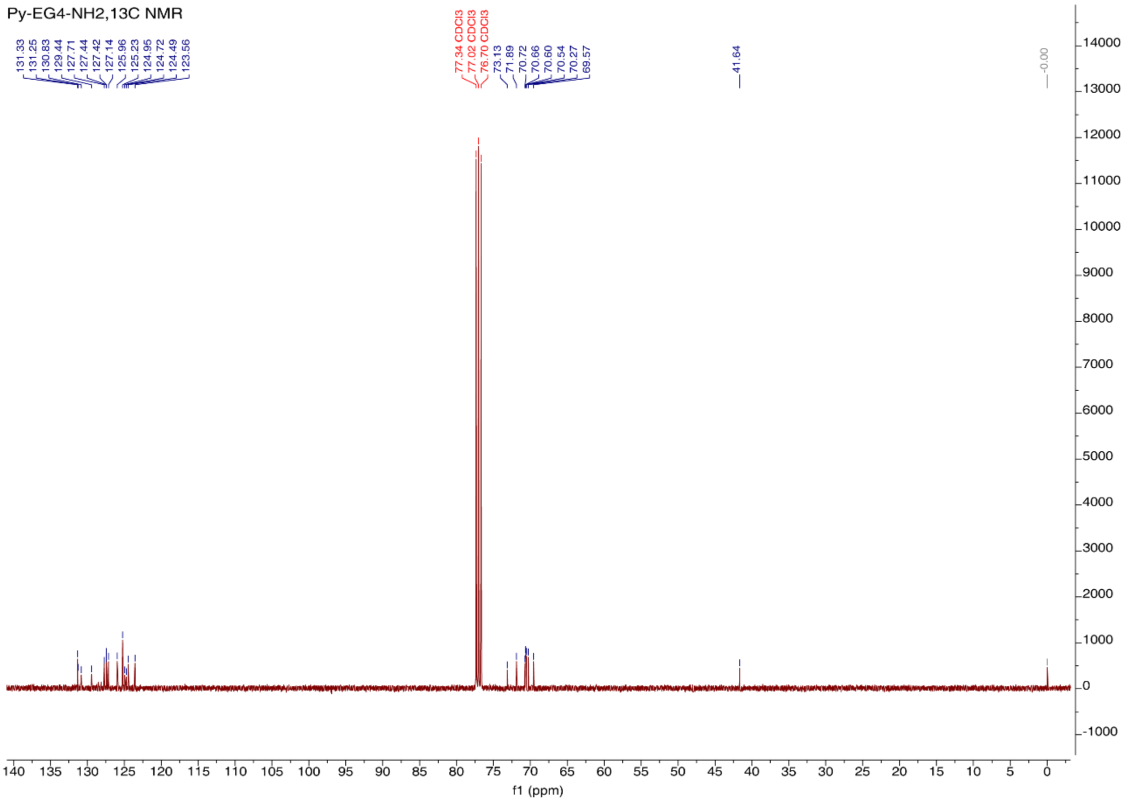

^{13}C NMR of Py-EG$_4$-NH$_2$ **5** in CDCl$_3$

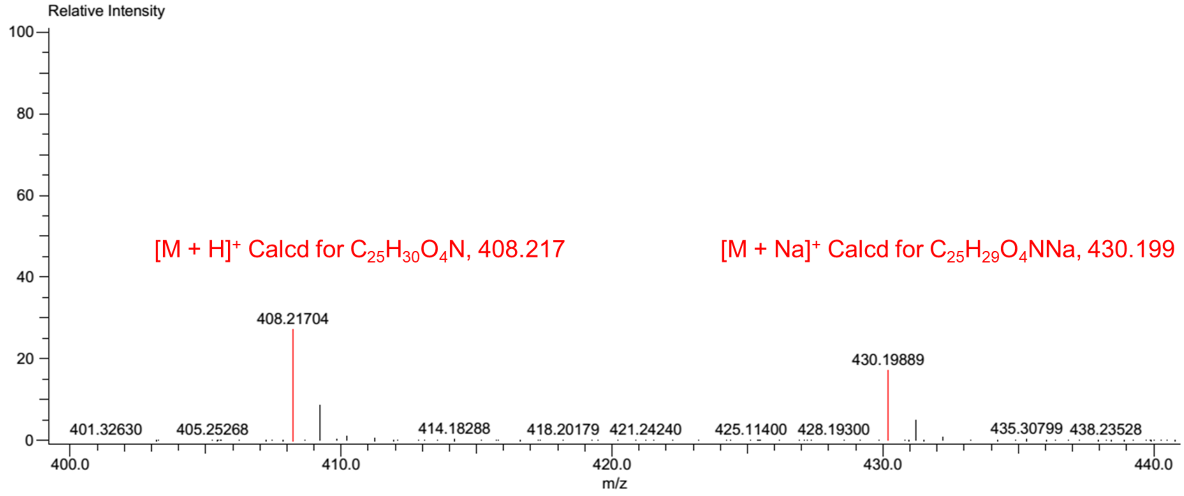

HR-MS of Py-EG$_4$-NH$_2$ **5**.
